# Adenine DNA methylation associated with transcriptionally permissive chromatin is widespread across eukaryotes

**DOI:** 10.1038/s41588-025-02409-6

**Published:** 2025-11-18

**Authors:** Pedro Romero Charria, Cristina Navarrete, Vladimir Ovchinnikov, Lan Xu, Luke A. Sarre, Victoria Shabardina, Ewa Ksiezopolska, Elena Casacuberta, David Lara-Astiaso, Arnau Sebé-Pedrós, Alex de Mendoza

**Affiliations:** 1 School of Biological and Behavioural Sciences, Queen Mary University of London, London, UK; 2 Centre for Epigenetics, Queen Mary University of London, London, UK; 3 Centre for Genomic Regulation (CRG), Barcelona Institute of Science and Technology (BIST), Barcelona, Spain; 4 Universitat Pompeu Fabra (UPF), Barcelona, Spain; 5 Institut de Biologia Evolutiva (CSIC-UPF), Barcelona, Spain; 6 Department of Haematology, University of Cambridge, Cambridge, UK; 7 Wellcome Trust—Medical Research Council Cambridge Stem Cell Institute, Cambridge, UK; 8 ICREA, Barcelona, Spain; 9 Wellcome Sanger Institute, Wellcome Genome Campus, Cambridge, UK

**Keywords:** Epigenetics, Epigenomics, Gene regulation

## Abstract

DNA methylation is a key regulator of eukaryotic genomes, most commonly through 5-methylcytosine (5mC). In contrast, the existence and function of *N*^6^-methyladenine (6mA) in eukaryotes have been controversial, with conflicting reports resulting from methodological artifacts. Nevertheless, some unicellular lineages, including ciliates, early-branching fungi and the alga *Chlamydomonas*, show robust 6mA signals, raising questions about their origin and evolutionary role. Here we apply Oxford Nanopore sequencing to profile 6mA at base-pair resolution across 18 unicellular eukaryotes representing all major supergroups. We find that robust 6mA patterns occur only in species that encode the adenine methyltransferase AMT1. Notably, 6mA consistently accumulates downstream of transcriptional start sites, positioned between H3K4me3-marked nucleosomes, indicating a conserved association with transcriptional activation. Our results support the idea that the last eukaryotic common ancestor had a dual methylation system, with transcription-linked 6mA and repressive 5mC, which has been repeatedly simplified in both multicellular and unicellular lineages through the loss of the AMT1 pathway.

## Abstract (web summary)

Long-read sequencing in 18 unicellular eukaryotes reveals that 6mA is widespread across eukaryotes and is enriched at transcriptionally permissive regions, which are also marked by H3K4me3.

## Main

Modified bases can change the way DNA is interpreted. The best studied modification is 5-methylcytosine (5mC), a widespread eukaryotic mark deposited by DNA methyltransferases (DNMTs)^1–3^, with functions ranging from transposable element (TE) control to gene regulation^4–6^. Other types of base modifications, like hydroxymethyluracil in dinoflagellates or the ‘base J’ in kinetoplastids, are lineage-restricted but abundant in some species^7,8^. In contrast, *N*^6^-methyladenine (6mA) has been reported across various lineages, including animals^9–12^, plants^13^, early-diverging fungi^14,15^, algae^16^ and ciliates^17,18^. However, global 6mA levels are often close to detection limits, with reports questioning its presence in animals, plants or yeast^19–23^. Challenges in 6mA quantification include antibody pull-down specificity issues, bacterial DNA contamination, RNA contamination and sequencing data artifacts^24,25^. Despite this, ciliates, early-diverging fungi and the alga *Chlamydomonas reinhardtii* exhibit high and orthogonally confirmed 6mA levels^14,19^. Thus, inadequate taxon sampling and conflicting results have limited our understanding of 6mA evolution.

**Fig. 1:**
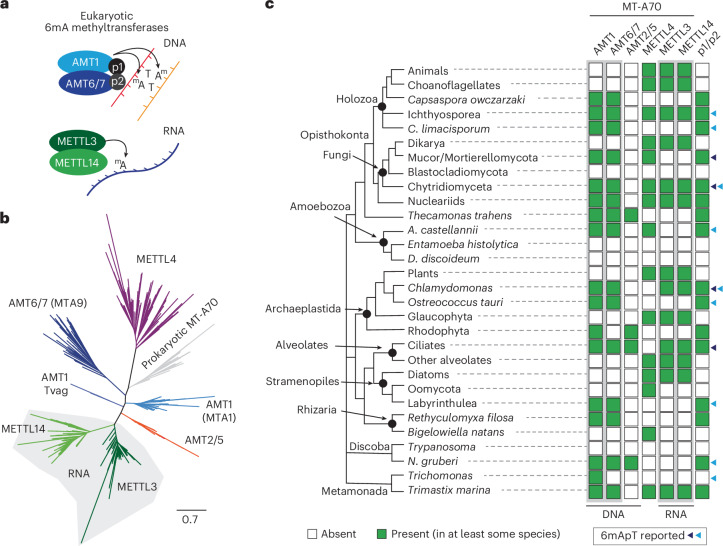
MT-A70 6-methyl-adenine methyltransferases origin and recurrent loss. **a**, Diagram of the known heterodimers formed by MT-A70 6mA methyltransferases. AMT1 and METTL3 are the active enzymes, whereas their partners are required but do not catalyze the reaction. p1 and p2 are described proteins from the ciliate *O. trifallax* that are required for AMT1 function^18^. **b**, Maximum-likelihood phylogenetic tree of MT-A70 6mA methyltransferases, including eukaryotic and prokaryotic sequences. Each clade is named according to previous references. *T. vaginalis* AMT1 paralogues highlighted as Tvag in the tree, yet their clustering as AMT1 sequences is shown in Extended Data Fig. 1a. **c**, Distribution of MT-A70 6mA methyltransferase family members across eukaryotes. For some lineages, the presence suggests that at least some species within the clade contain them. Dark triangles depict species for which 6mApT levels were reported previously, and pale blue triangles are species covered in this study. Phylogenetic relationships across these species are based on consensus in the field^35,82^. Source data

A mechanistic understanding of genomic 6mA in eukaryotes comes from research in ciliates. In ciliates, the 6mA methyltransferases belong to the MT-A70 family, unrelated to DNMTs but sharing structural homology with the m6A RNA methyltransferases, METTL3 and METTL14 (refs. ^26–28^). AMT1 (known as MTA1 in *Oxytricha trifallax*) is the main 6mA writer in ciliates, while accessory enzymes from families AMT6/7 (MTA9 in *O. trifallax*) are dispensable and lack catalytic activity^18,29^. However, AMT6/7 forms a heterodimer with AMT1 during DNA engagement, akin to the METTL3 and METTL14 heterodimer (Fig. 1a)^30^. Additionally, DNA-binding proteins p1 and p2 have a role in targeting genomic DNA by forming a multimeric complex with AMT1 (refs. ^18,29^). In ciliates, early-diverging fungi and the alga *C. reinhardtii*, 6mA is confined to ApT dinucleotides^14,16–18,28^. It is suggested that AMT1 binds hemimethylated ApT sites, as does DNMT1, thereby maintaining 5mC on CpG dinucleotides^31^. In ciliates and *C. reinhardtii*, 6mA is found in internucleosomal linker DNA^16–18,32^, particularly enriched downstream of the transcriptional start site (TSS) of active genes. This association with transcription contrasts the proposed role of 6mA in silencing TE in animals^11,12,22^. Here 6mA may be evolutionarily flexible, like 5mC, which in different eukaryotes is restricted to transposon silencing or gene bodies^4–6^. However, the lack of data from many eukaryotic lineages limits our ability to reconstruct 6mA evolution and function.

To clarify these uncertainties, in this work, we study the evolutionary history of MT-A70 methyltransferases and we find that the AMT1 pathway can be traced back to the last eukaryotic common ancestor (LECA). Then, using Oxford Nanopore modified base calling, previously validated for identifying this base modification in eukaryotes^33^, we detect consistent 6mA patterns in representatives from the major eukaryotic supergroups.

## Results

### AMT1 is ancestral but has been repeatedly lost in eukaryotes

**Extended Data Fig. 1:**
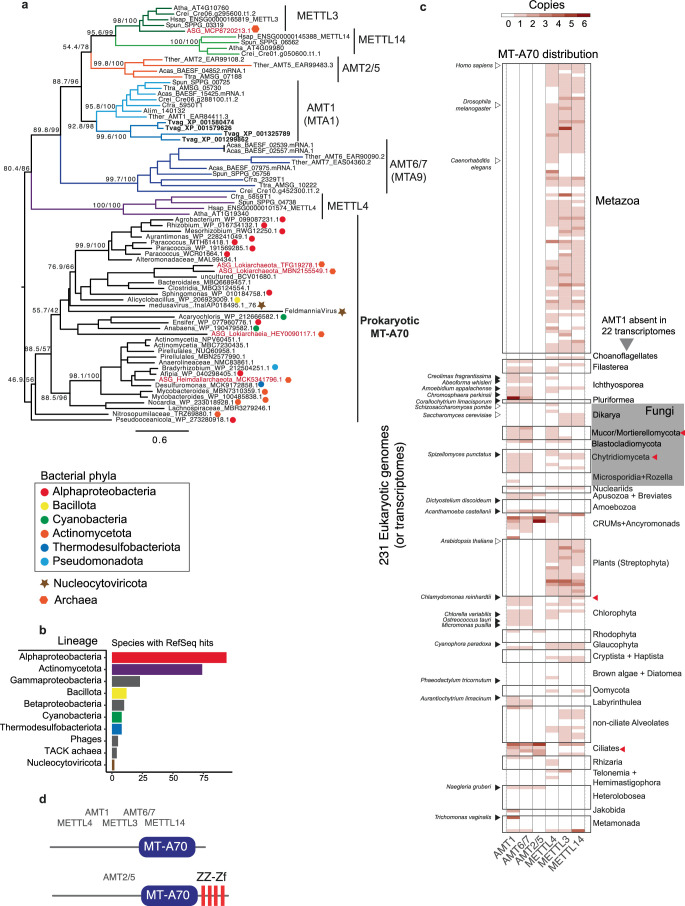
Bacterial outgroups, structure and distribution of MT-A70. **a**, Maximum likelihood phylogenetic tree with selected species from Fig. 1b highlighting the prokaryotic outgroups, color coded by bacterial lineage (for those bacteria with genus/species defined). *Trichomonas vaginalis* AMT1 sequences are highlighted in bold, whereas Asgardarchaeota are highlighted in red. Nodal supports represent SH-aLRT and ultrafast bootstrap values. **b**, Taxonomy count of MT-A70 BlastP searches against NCBI RefSeq_select database (January 2024). **c**, Heatmap showing the number of MT-A70 family members across a collection of 231 eukaryotic species covering a wide scope of known diversity. Classification obtained from a phylogenetic tree in Fig. 1b. Black triangles are the species sequenced in this study, and red arrows indicate lineages/species for which 6mA has been previously confirmed at robust high levels, white triangles indicate species for which 6mA is contested. **d**, Domain architectures of eukaryotic MT-A70 families, defined with Pfam domains MT-A70 (PF05063) and ZZ (PF00569). Source data

To clarify the origins and evolution of MT-A70 methyltransferases, we examined 231 eukaryotic genomes and transcriptomes (Supplementary Table 1). Additionally, we searched for noneukaryotic MT-A70 homologs, whose origins remain unclear^1,26^. The resulting MT-A70 phylogenetic tree mirrors the following six established eukaryotic families: AMT1, AMT6/7, AMT2/5, METTL3, METTL14 and METTL4 (Fig. 1b)^26,28^. Notably, prokaryotic sequences form a distinct clade, suggesting a bacterial origin for MT-A70 inherited early in eukaryotic evolution (Fig. 1b and Extended Data Fig. 1). MT-A70 presence in prokaryotes is confined to select eubacterial phyla, with few archean genomes harboring it, typically in a single copy per species (Extended Data Fig. 1a,b). Asgardarchaeota sequences cluster within prokaryotes rather than as sister to eukaryotes, arguing against an archaeal donor. Only one Asgardarchaeota of the 302 genomes searched contains a METTL3-like sequence, likely from metagenomic assembly contamination (Extended Data Fig. 1a). Unlike the more widespread bacterial-type Dam 6mA methyltransferase, MunI-like 6mA MT-A70 in bacteria is less diverse^1^. The monophyletic clustering of bacterial sequences supports a single origin of eukaryotic MT-A70 from an unidentified bacterial donor, or, alternatively, an eukaryotic origin followed by a single lateral gene transfer to prokaryotes, which is less parsimonious. This rules out recurrent transfers from prokaryotes to eukaryotes.

We found that all six MT-A70 families are present in most eukaryotic supergroups (Fig. 1c), suggesting that they duplicated before LECA. The RNA-associated METTL3 and METTL14 co-occur across the species phylogeny, in a pattern similar to the DNA-associated AMT1 and AMT6/7, suggesting that both enzymatic pairs have conserved heterodimer associations across eukaryotes (Fig. 1c and Extended Data Fig. 1c). AMT2/5 is less common, often co-occurring with AMT1, hinting at a potential partnership but one in which AMT2/5 is dispensable. Notably, AMT2/5 is exceptional as it possesses C-terminal ZZ zinc finger domains of unknown function (Extended Data Fig. 1d)^26,28^. In contrast, METTL4 does not co-occur with any MT-A70 families, suggesting that it does not form heterodimers (Fig. 1c and Extended Data Fig. 1c). METTL4 is believed to methylate mitochondrial DNA or small RNAs, but its role is currently not well understood^22,34^. The nematode *Caenorhabditis elegans* METTL4 ortholog (known as DAMT-1) has been suggested as a genomic DNMT^10^, yet this is contested^20^. Beyond the MT-A70 methyltransferases, the proteins p1 and p2 exclusively co-occur with AMT1 (Fig. 1c), indicating an ancestral link with the AMT1 pathway^18^.

Hence, the enzymes responsible for RNA and DNA 6mA exhibit ancient origins, yet their evolution appears largely decoupled to each other in eukaryotes. Notably, AMT1 is consistently found in species with documented robust 6mA levels, such as ciliates, *C. reinhardtii* and early fungi^14,16–18^. Although exceptions exist, AMT1 co-occurs with AMT6/7 and p1/p2 partners (for example, *Trichomonas vaginalis*).

### 6mA correlates with transcription in AMT1-encoding species

**Fig. 2:**
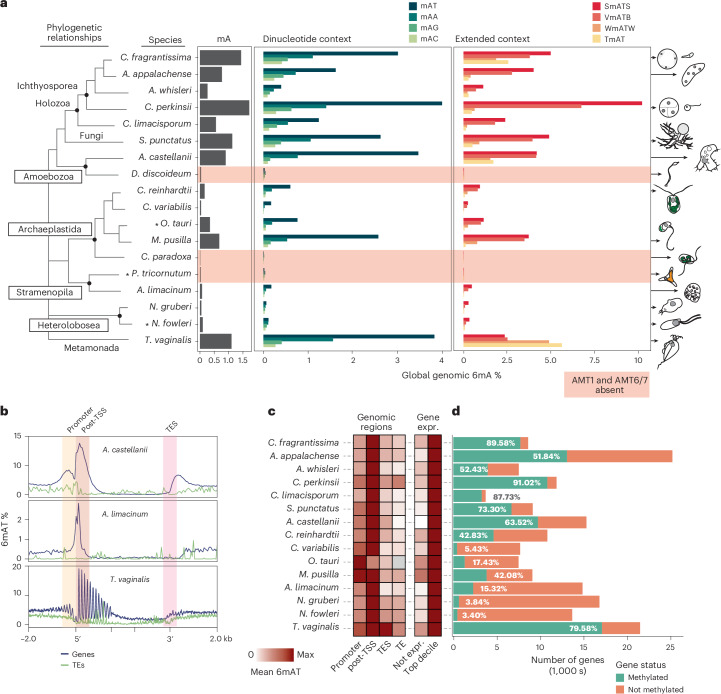
Nanopore sequencing reveals widespread 6mA in AMT1-encoding eukaryotic genomes. **a**, Global methylation levels of 6mA in different sequence contexts across 18 eukaryotes representing deep branching points of eukaryotic diversity. S stands for C or G, W stands for A or T, V stands for A,C,G and B stands for T,C,G. Species with red shade do not encode for AMT1 or its partner AMT6/7. Asterisks indicate Oxford Nanopore datasets obtained from NCBI/ENA and silhouettes represent the shapes of the species. **b**, Average 6mA levels in the AT context over three example species, including genes (blue) and TEs (green; >1,000 bp). Thus, 5′ and 3′ ends of the features are marked with dashed lines, the first and last 1,500 bp are unscaled, and the shades indicate the regions used in **c**. **c**, Heatmap displaying average 6mA levels across various genomic regions, including the 500-bp downstream of the post-TSS, the 500 bp centered around the TES and TEs. Also, for gene-body 6mA levels for nonexpressed genes and the top decile of highly expressed genes, see full values in Extended Data Fig. 3. Gray cell indicates missing data, as *O. tauri* has too few repeats. **d**, Number of genes methylated in each genome (see Methods for thresholds). TES, transcriptional end site. Source data

Based on the AMT1 distribution, we searched for eukaryotic lineages with detectable genomic 6mA. To avoid bacterial contamination, we focused on species grown axenically. Our selection included 15 species encoding AMT1 and three species lacking AMT1 orthologs as negative controls. Our sampling of ichthyosporeans, *Corallochytrium limacisporum*, a chytrid fungus, amoebozoans, chlorophytes, a glaucophyte, stramenopiles, heteroloboseans and a metamonada spans major eukaryotic branching groups^35^. Genomic DNA was sequenced using Oxford Nanopore R9.4.1 technology for 15 species, while equivalent public data were used for three more^36–38^. Using the Guppy software with the ‘all-context’ Rerio model, we obtained maps for 6mA and 5mC in all species. Global methylation levels varied substantially across species, with the lowest levels observed in species lacking AMT1 (Fig. 2a).

**Extended Data Fig. 2:**
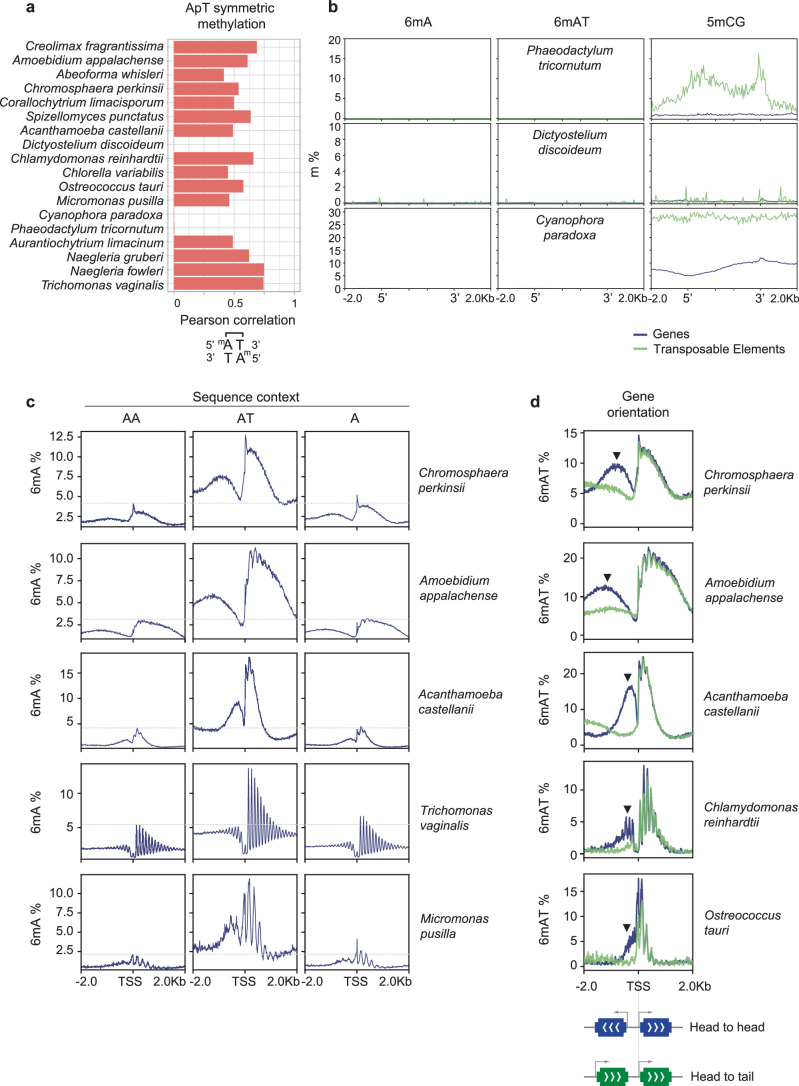
Patterns of 6mA distribution in eukaryotes. **a**, Global correlation of 6mA levels in ApT dinucleotides. Pearson’s correlation was calculated for ApT positions with at least 10x coverage on each strand. Please note the lack of correlation in AMT1-lacking species. **b**, Average methylation levels in 6mA and 5mC for three species that lack AMT1 or AMT6/7. *Dictyostelium discoideum* also lacks DNMTs and has previously been reported to lack 5mC; thus, the signal is likely noise (also driven by the low number of annotated TEs—88). Borders of genes and TEs are represented by the 5’ and 3’ sites. The first 1,500 bp post-TSS and before the TES are unscaled. **c**, Average 6mA levels around the transcriptional start site (TSS) in five species with high 6mA levels (Fig. 2a), divided by sequence context. AT dinucleotides are the preferentially methylated context, but the AA context also gets residual methylation on the same regions, yet on average less than the total 6mA methylation. The dashed line highlights the max reached by AA methylation. **d**, Average 6mA level plot around the TSS in species with marked ‘promoter’ methylation patterns. Genes with a head-to-head orientation are displayed in blue and genes with a head-to-tail orientation are displayed in green. The black arrow shows the artifactual ‘promoter’ peak driven by head-to-head-oriented genes. Source data

Because AMT1 preferentially methylates ApT dinucleotides in ciliates^14,16,17^, we assessed global 6mA levels across various sequence contexts. In all AMT1-encoding species, AT was the main methylated context, although AA also showed appreciable levels (Fig. 2a). ApT sites displayed symmetric methylation across all AMT1-encoding species (Extended Data Fig. 2a). In contrast, AMT1-lacking species had negligible difference among dinucleotide contexts, with the AA dinucleotide being the most highly enriched (Fig. 2a and Supplementary Fig. 1). Notably, *C. reinhardtii* and early-diverging fungi exhibit higher methylation in the VATB context (V = C/G/A, B = C/G/T)^14,19^, whereas in fungi the TAT trinucleotide showed the lowest methylation^14^. Thus, we calculated global levels for three 4-mer combinations and the TAT trinucleotide to discern AT flanking specificity. Across all AMT1-containing species SATS (S = C or G) exhibited the highest methylation, while TAT and WATW (W = A or T) showed the lowest (Fig. 2a). The VATB context displayed intermediate levels, suggesting that cytosine and guanine flanking dictate methyltransferase specificity (Fig. 2a). An exception was the metamonad *T. vaginalis*, which encodes four AMT1 paralogues but lacks AMT6/7, p1 or p2 (Fig. 1c). In *Trichomonas*, WATW and TAT were more enriched than SATS (Fig. 2a), indicating the signal reflects distinct methyltransferase preferences and showing that these patterns are not due to Oxford Nanopore base-calling bias.

**Extended Data Fig. 3:**
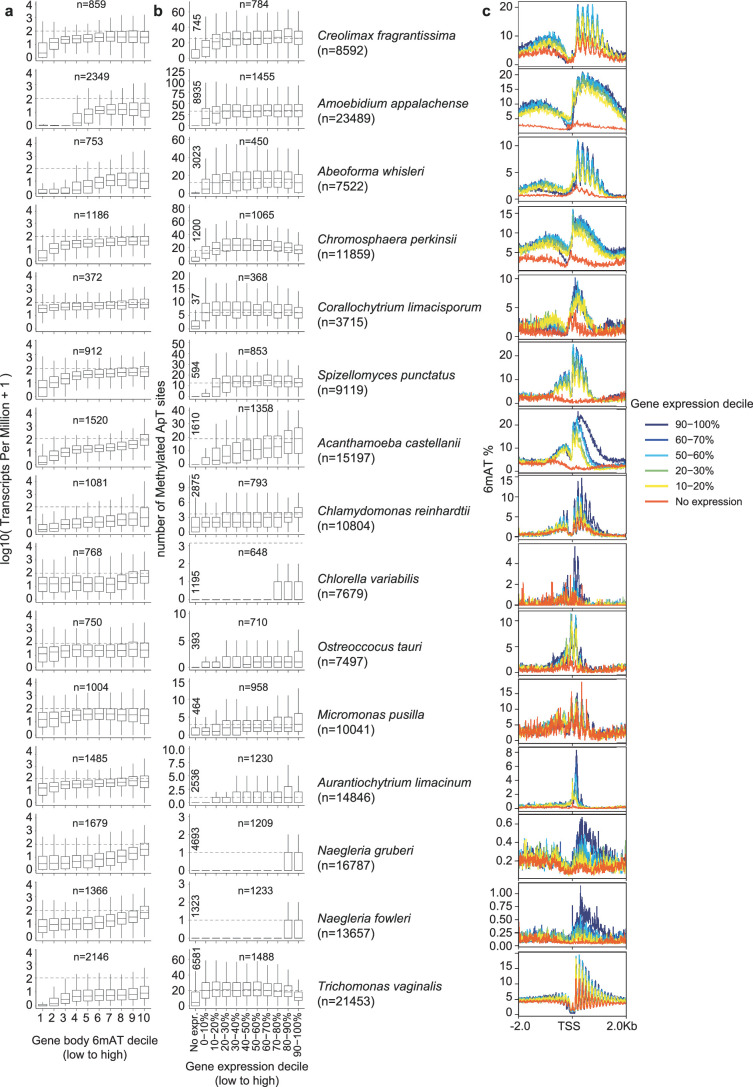
6mA correlates with higher transcription across eukaryotes, encoding AMT1. **a**, Distribution of transcription levels of genes divided into ten deciles sorted by the gene body 6mA levels in the AT context. Number of genes in deciles shown on top. Boxplots show the median (centerline), interquartile range (box), and the smallest and largest values within 1.5× IQR (whiskers). **b**, Distribution of methylated AT sites (>10% 6mA) in gene bodies sorted in ten deciles by transcriptional level (numbers per deciles shown on top), where not expressed genes (TPM < 1) are all shown separately (total number on no. expr. category). ‘n = ’ in parenthesis indicates the total amount of genes shown for each species. Boxplots show the median (centerline), interquartile range (box), and the smallest and largest values within 1.5× IQR (whiskers). **c**, Average 6mA levels in the AT context around the TSS for genes sorted by transcriptional levels as in **b**. Decile of expression color code shown in legend. TPM, Transcripts per Million. Source data

**Extended Data Fig. 4:**
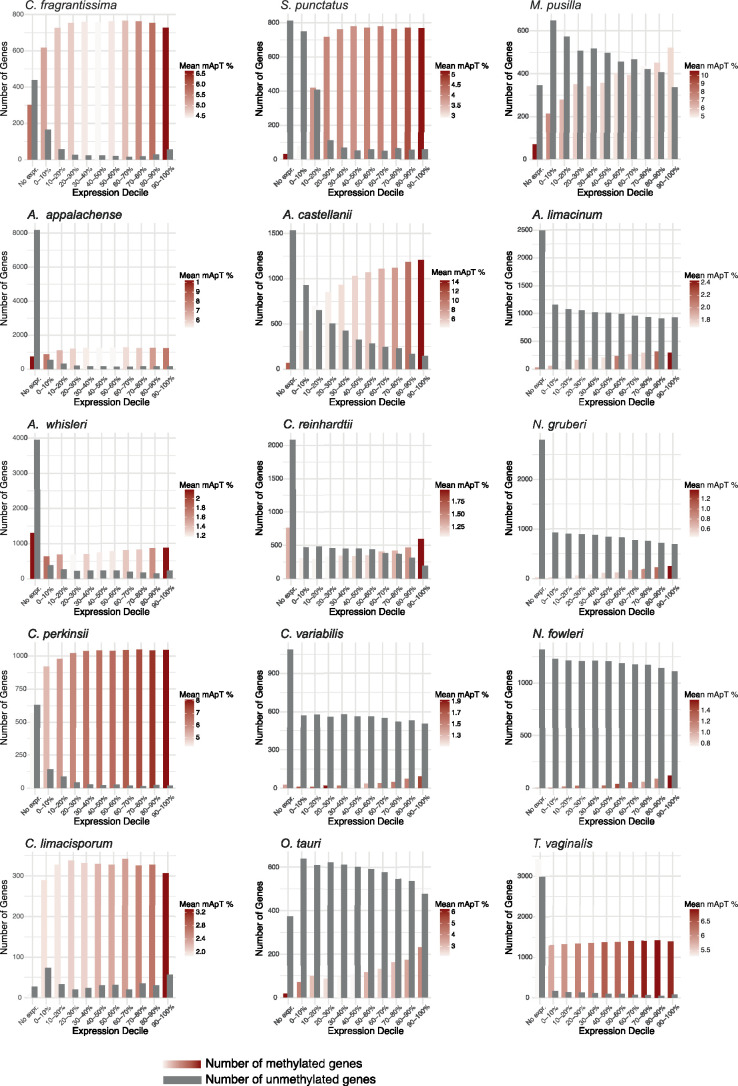
Correlation of 6mA with transcription varies among eukaryotes encoding AMT1. The figure displays the number of 6mA-methylated versus unmethylated genes across transcriptional deciles. For methylated genes, bars are colour-coded to reflect the average 6mA at ApT percentage, while unmethylated genes are represented by grey bars. Source data

We then examined the genomic distribution of 6mA. In species without AMT1, 6mA appeared uniformly across genes and TEs (Extended Data Fig. 2b), consistent with the background noise. Conversely, in AMT1-encoding species, a peak of AT methylation emerged proximal to the TSS (Fig. 2b,c). AA methylation mirrored these patterns, suggesting that AA methylation might be an off-target substrate of AMT1 (Extended Data Fig. 2c). Some species displayed a subtle 6mA enrichment in promoter regions (upstream of the TSS; Fig. 2c)^16^, which proved to be an artifact arising from the head-to-head orientation of upstream genes (Extended Data Fig. 2d). Neither transcriptional end sites nor TEs exhibited enrichment in 6mA (Fig. 2c). Genic 6mA correlated with gene transcriptional activity (Fig. 2c). Across all species, silent genes presented lower 6mA levels compared to highly expressed genes, which also harbored more methylated ApT sites (Fig. 2c and Extended Data Figs. 3 and 4). The association with transcription was quite variable; for instance, *Acanthamoeba castellanii* exhibited a gradual link with transcriptional levels, while in ichthyosporeans or prasinophytes all genes above a specific transcriptional threshold presented uniform 6mA levels (Extended Data Figs. 3 and 4). This diversity was also evident in the total number of genes displaying methylation in each genome (Fig. 2d and Supplementary Fig. 2). The ichthyosporean *Chromosphaera perkinsii* exhibited 92% of genes with 6mA, whereas both *Naegleria* species displayed detectable methylation in only 3% genes (Fig. 2d). Notably, most amino acid positions on the AMT1 sequences were under strong purifying selection (Supplementary Table 3), suggesting that the variation in targeting mechanisms rather than in the AMT1 protein itself is responsible for its differential usage, perhaps underlying distinct promoter architectures^39^.

### Stage-specific transcription occurs independent of 6mA

**Fig. 3:**
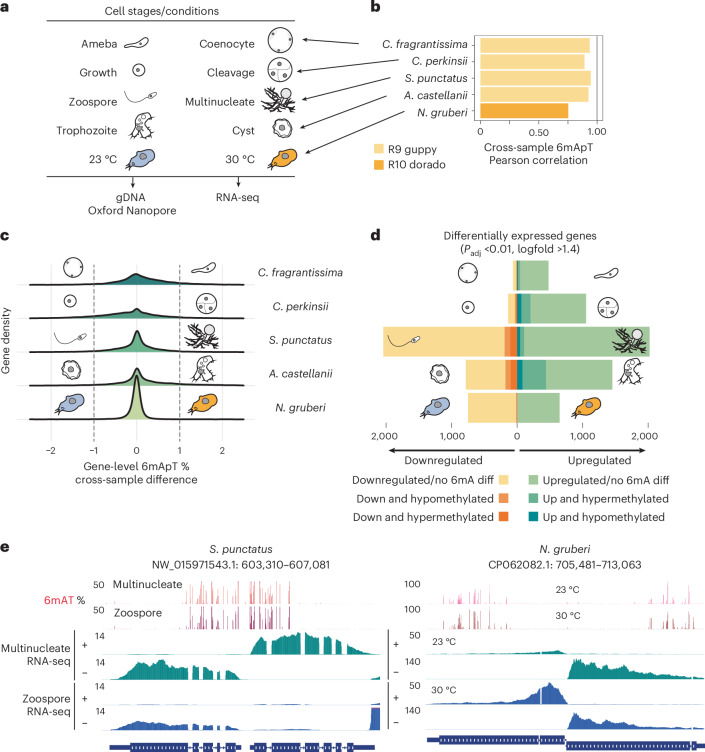
Stable 6mA patterns correlate weakly with stage-specific transcriptional changes. **a**, Collection of species and stages/treatments profiled in this study. **b**, Global 6mA correlation levels calculated for all ApT sites in each stage. All species were profiled using R9.4.1 ONT chemistry and processed with Guppy, except for *N. gruberi*, which was profiled using R10.4.1 ONT chemistry and processed with Dorado. **c**, Distribution of gene-body 6mA percentage differences between two stages across all five species comparisons. A threshold of ≥1% 6mA delta was used to classify a gene as hypermethylated or hypomethylated. **d**, Number of differentially expressed genes across stages, divided by 6mA methylation change categories (no change, hypermethylated, hypomethylated). **e**, Genome browser snapshots showing differentially transcribed genes alongside stable 6mA levels in *S. punctatus* and *N. gruberi*. RNA-seq values are normalized to CPM and displayed separately for each strand. ONT, Oxford Nanopore Technologies; CPM, counts per million. Source data

**Extended Data Fig. 5:**
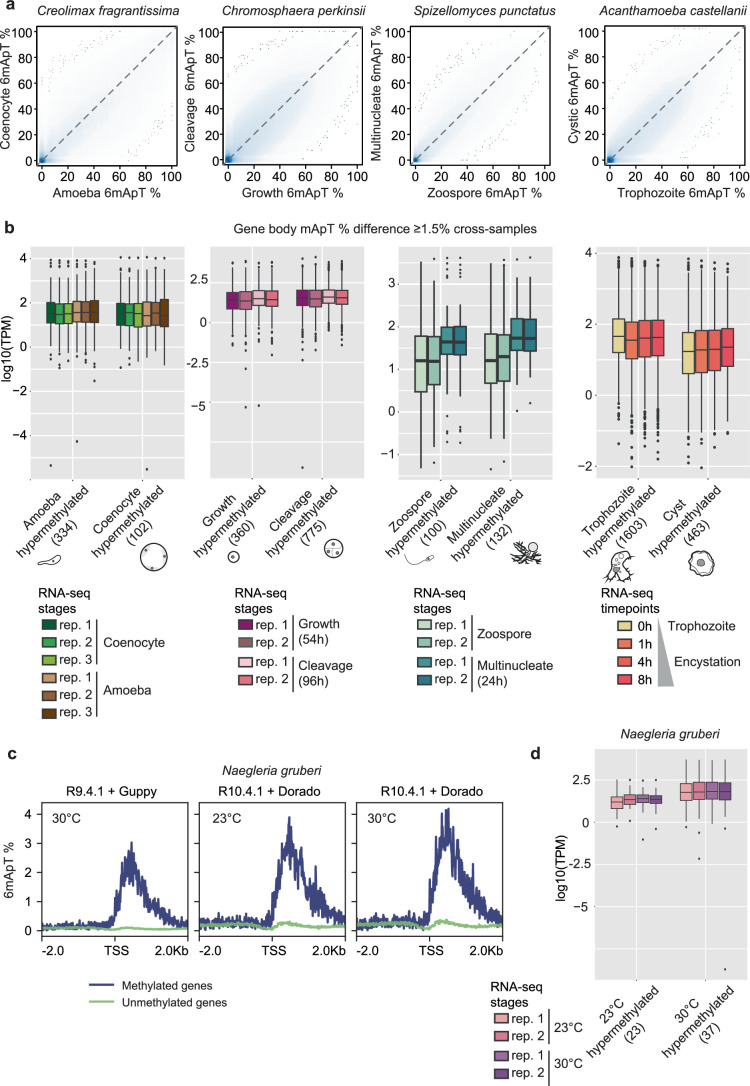
6mA methylation is modestly responsive to transcriptional changes. **a**, Comparison of 6mA levels in ApT dinucleotides across two cell stages in four species. Only ApT sites with at least 10x coverage in both samples are shown. **b**, Boxplot displaying the transcriptional levels of genes with the variable 6mA levels (≥1.5% 6mApT gene difference) across cell stages. Total number of genes in each category shown in the x-axis labels. Each RNA-seq sample is color coded according to each species legend. Boxplots show the median (centerline), interquartile range (box), and the smallest and largest values within 1.5 × IQR (whiskers); points outside this range are plotted as outliers. **c**, Average methylation levels on methylated and unmethylated genes in *N. gruberi*. Methylated genes were defined using R9.4.1 and Guppy as base modification algorithm, whereas 23 °C and 30 °C samples were sequenced using R10.4.1 pore chemistry and 6mA calls done using Dorado. **d**, Boxplot displaying the transcriptional levels of genes with the variable 6mA levels (>1.5% 6mApT gene difference) across *N. gruberi* temperature treatments. Boxplots show the median (centerline), interquartile range (box), and the smallest and largest values within 1.5× IQR (whiskers); points outside this range are plotted as outliers. Source data

We then tested how variable 6mA is upon transcriptional changes. We chose the amoebozoan *A. castellannii*, the fungus *Spizellomyces punctatus*, the ichthyosporeans *Creolimax fragrantissima* and *C. perkinsii* because these have different cell stages that can be isolated in culture^40–43^ (Fig. 3a), high 6mA levels and distinct associations between 6mA and transcriptional level in the steady state (Extended Data Fig. 3). We also collected matched transcriptomic and DNA samples for *Naegleria gruberi* grown at 23 °C and 30 °C. All species presented static 6mA methylomes at ApT and gene-level resolution, where most genes displayed minimal 6mA differences (Fig. 3b,c and Extended Data Fig. 5a,c). In *N. gruberi*, the 644 genes called methylated using R9 nanopore chemistry, showed comparable 6mA levels at 23 °C and 30 °C, despite using R10 chemistry and a distinct analytical pipeline (Extended Data Fig. 5c). When we computed the total number of differentially expressed genes across stages and overlapped these with genes exhibiting >1% change in 6mApT levels along the gene body, we observed that thousands of genes change expression without any detectable 6mA alterations (Fig. 3d). In *A. castellanii*, 24% of the trophozoite upregulated genes were also hypermethylated in this stage, following a positive correlation with transcriptional change, yet the rest of trophozoite upregulated genes did not show methylation changes and 5.57% were hypomethylated, implying a limited link between 6mA and transcriptional change. In the *S. punctatus* zoospore, some genes were almost completely silenced in one stage yet retained high 6mA levels as in the transcribed stage (Fig. 3e). Further analysis of genes with variable 6mA (>1.5%) showed no correlation with transcriptional changes (Extended Data Fig. 5b,d). Although 6mA is associated with steady-state transcription in all AMT1-encoding eukaryotes, its link to dynamic transcription is weak, suggesting that 6mA reprogramming is dispensable for transcriptional change.

### 5mC and 6mA mark different functions in eukaryotes

**Fig. 4:**
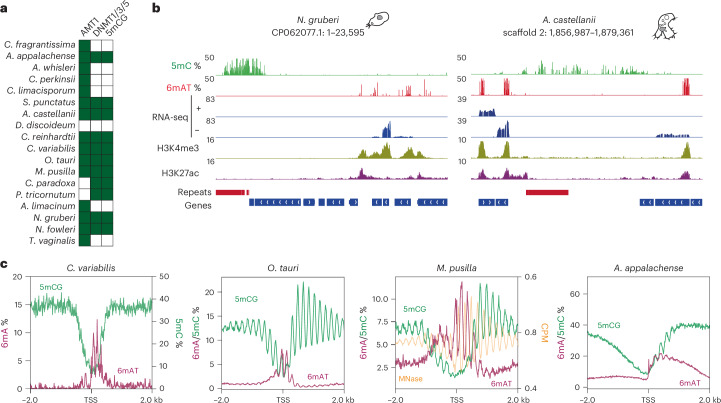
6mA and 5mC are compartmentalized in eukaryotic genomes. **a**, Distribution of DNMTs and the presence of 5mC in the CG context across the species in our dataset. Dark green indicates presence, while white indicates absence. **b**, Genome browser snapshots of *N. gruberi* and *A. castellanii* showing 6mA enrichment downstream of expressed genes and 5mC demarcating transcriptionally silent repetitive regions. RNA-seq (strand-specific) and ChIP–seq data are scaled using CPM. **c**, Average methylation levels centered at the TSS in species that have 5mC gene-body methylation. For *C. variabilis*, 6mA and 5mC axes are shown separately. For *M. pusilla*, MNase data are shown to highlight the anticorrelation with both 5mC and 6mA peaks, as CPM. MNase, micrococcal nuclease.

**Extended Data Fig. 6:**
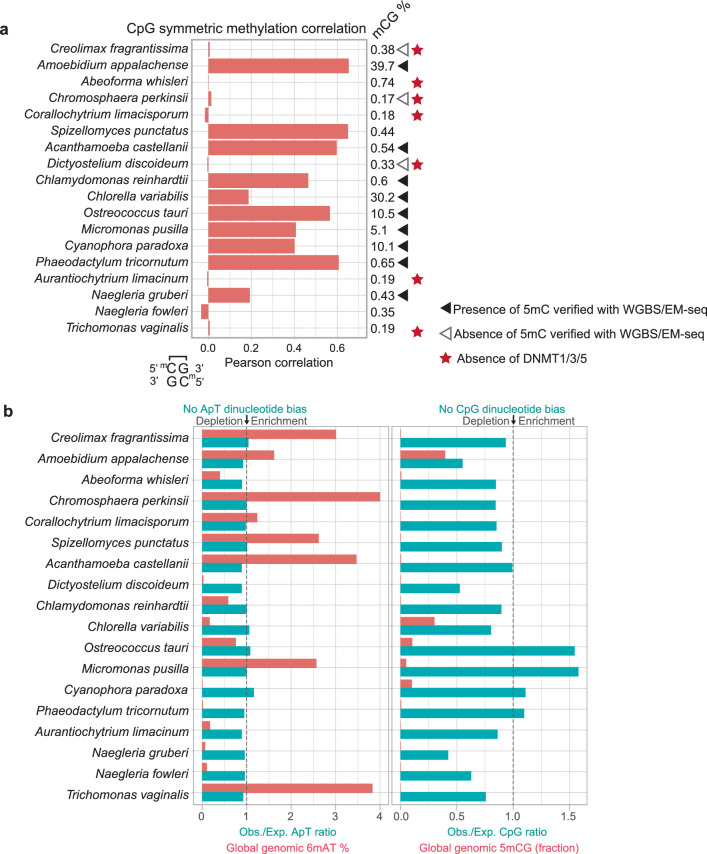
6mA methylation is not associated with a depletion of AT dinucleotides. **a**, Global correlation of 5mC levels at CpG dinucleotides. Pearson’s correlation was calculated for CpG positions with at least 10× coverage on each strand, noting the lack of correlation in species lacking DNMT1/3/5. Global 5mCG levels were estimated using Oxford Nanopore data. Black triangles indicate species for which WGBS/EM-seq data have confirmed the presence of this modification, while white triangles indicate confirmed absence. **b**, The global genomic 6mA levels in AT context (red) are shown next to the observed versus expected ApT dinucleotide ratios (turquoise) in each of sampled genomes. An observed-to-expected ratio of 1 indicates that ApT dinucleotides are present at the expected ratio given the genomic AT%, whereas <1 levels indicate depletion and>1 levels indicate enrichment. For comparison, global genomic 5mC levels (shown as a fraction with maximum being 1.0 in red) in the CG context are shown next to the observed versus expressed CG dinucleotide context ratio (turquoise) for the same set of species. Source data

As 5mC is the other widespread form of DNA methylation in eukaryotes, we examined its relationship with 6mA within our species set (Fig. 4a). We found diverse scenarios—species exhibited nondetectable 5mC levels alongside high 6mA (for example, *C. limacisporum*), while others exclusively harbored 5mC (*Phaeodactylum tricornutum*). Some species presented both modifications, and *Dictyostelium discoideum* displayed neither (Fig. 4a). Negligible 5mC levels and the absence of CpG symmetry detected with nanopore matched previous results from enzymatic methyl-sequencing and whole-genome bisulfite sequencing in these species^44,45^ (Extended Data Fig. 6a). This variation suggests that 6mA is not evolutionarily linked to 5mC, corroborating observations in early-diverging fungal lineages^14^.

The recurrent loss of 5mC in eukaryotes is partly attributed to its mutagenic effects^46^. Species with elevated 5mC levels, like vertebrates, often have fewer CpG dinucleotides than expected by chance, because deamination of 5mC into thymine depletes CpGs over evolution^47^. In contrast, animals with low or no 5mC lack this compositional bias^48^. Using a similar approach, we calculated observed-to-expected ApT ratios across our species. Global 6mAT levels did not correlate with ApT dinucleotide depletion, with a relatively constant ratio across species (Extended Data Fig. 6b). Notably, CpG depletion was observed in species with very low 5mCG levels (for example, *Naegleria*), while prasinophytes exhibited the opposite trend, with high 5mCG levels and enriched CpG composition (Extended Data Fig. 6b) as previously reported^3^. These findings suggest that neither 5mC nor 6mA substantially shapes genomic base composition across the vast diversity of eukaryotes, in contrast with prior observations for 5mC in animals and plants.

**Extended Data Fig. 7:**
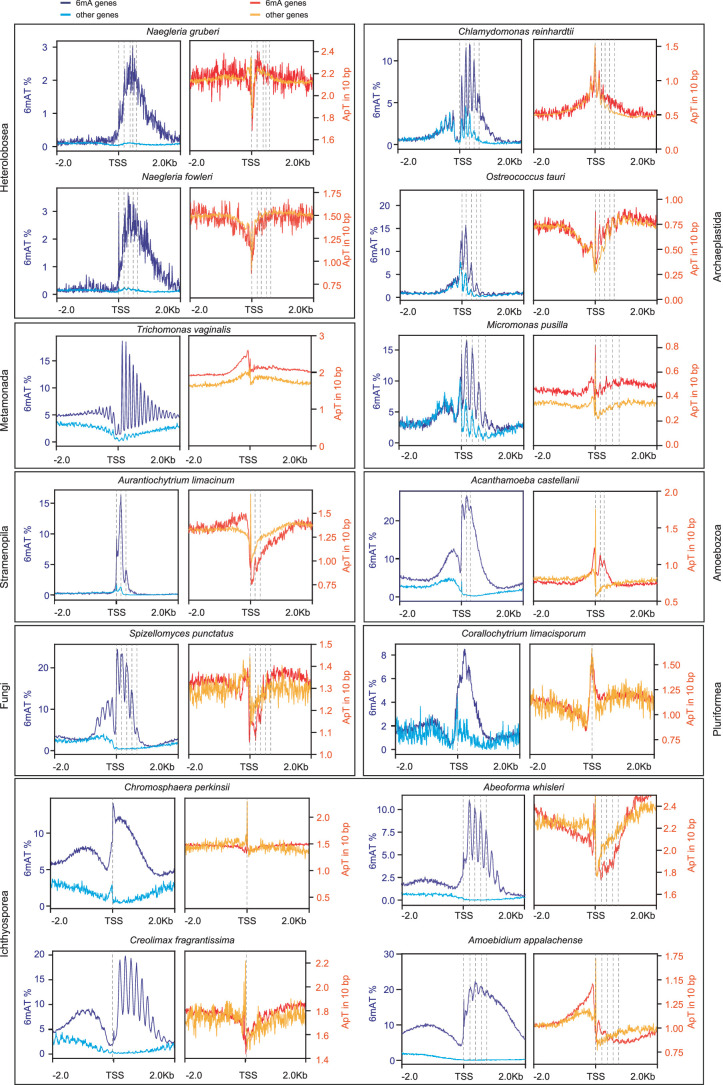
6mA rarely recapitulates the ApT dinucleotide composition downstream of the TSS. Average 6mA levels in the AT context shown side by side to average ApT content in genes separated by their methylation status. Methylated genes are shown in dark blue (6mA plot) and dark red (ApT content plot), whereas unmethylated genes are shown in pale blue (6mA plot) and orange (ApT content plot). Windows for averaging are 10 bp long. Enrichment of ApT in the TSS is likely driven by lack or inaccurate UTR annotations in many gene models, with 5’ end of genes being the start codon ‘ATG’. Gray dashed lines indicate the TSS position and the 6mApT peaks in each species.

One possibility is that 6mA mutagenic effects are most evident in regions where it is enriched, particularly post-TSS. Similarly, in invertebrates with 5mC in gene bodies, methylated genes show lower CpG ratios than unmethylated ones^49,50^. We grouped genes by the presence or absence of methylated ApTs and compared local ApT density. In most species, 6mA showed no clear association with ApT composition (Extended Data Fig. 7). In some species, methylated genes had higher ApT density than unmethylated ones, opposite to patterns seen with 5mC. In early-diverging fungi, 6mA-methylated genes are depleted for TAT motifs. We confirm this in the fungus *S. punctatus*, but not across other eukaryotes (Supplementary Fig. 3). Thus, the recurrent loss of 6mA in eukaryotes is unlikely to be driven by mutagenic effects, which appear limited.

**Extended Data Fig. 8:**
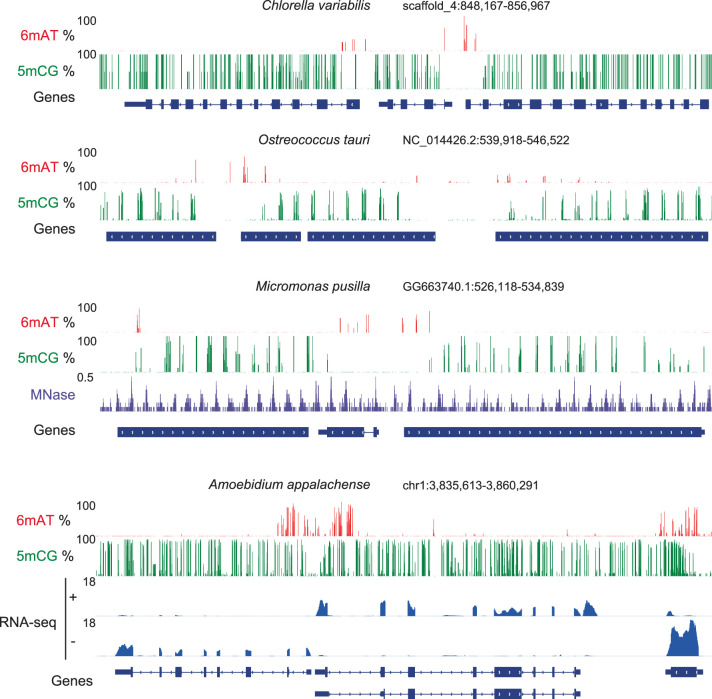
5mC in gene bodies coexists with post-TSS 6mA. Genome browser snapshots of four species with 5mCG on gene bodies. MNase and RNA-seq data are normalized using reads per million (RPM).

In species with both 6mA and 5mC, we observe two main patterns. In *Acanthamoeba*, *Naegleria*, *Spizellomyces* or *Chlamydomonas*, as in the previously reported early-branching fungi^14,51^, 5mC is confined to transposons while 6mA marks expressed genes (Fig. 4b), suggesting opposing links to transcription. Nevertheless, in several eukaryotes, 5mC is also found within gene bodies, including animals and plants^52,53^, but also the chlorophytes and the ichthyosporean *Amoebidium appalachense* in our dataset^3,45^. In this second pattern, 6mA predominantly occupies the five prime ends of the gene body, while 5mC peaks when 6mA diminishes (Fig. 4c). In all four species, there is a distinct periodic 6mA pattern suggestive of internucleosomal positioning (Fig. 4c). We used micrococcal nuclease sequencing data for *Micromonas pusilla* to confirm that 6mA peaks are anticorrelated with nucleosome positioning (Fig. 4c and Extended Data Fig. 8)^3^. In *Amoebidium*, both modifications coexist in the first three or four nucleosome territories downstream of the TSS (Fig. 3c and Extended Data Fig. 8). In the case of *Micromonas* and *Ostreococcus*, nanopore data align with the reported pattern of internucleosomal 5mC distribution along the gene bodies of prasinophytes^3^. The original study noted that 5mC is weakly linked to transcription in prasinophytes, suggesting a compaction role in these microalgae with very small genomes (for example, *Ostreococcus tauri*, 12.6 Mb)^3^. Notably, 6mA replaces 5mC in the first nucleosomes downstream of the TSS of active genes, suggesting a role in maintaining transcriptional permissiveness, as inactive genes show less 6mA (Extended Data Figs. 3 and 4).

### Nucleosomes with H3K4me3 colocalize with 6mA

**Fig. 5:**
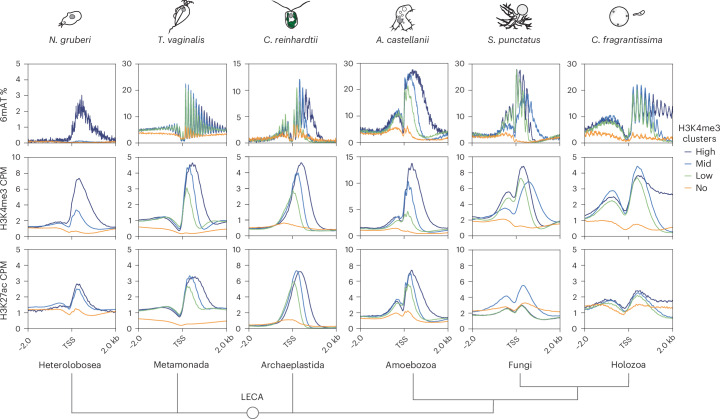
Nucleosomes with H3K4me3 modification recapitulate 6mA patterns. Average 6mA (on ApT context), H3K4me3 and H3K27ac across five eukaryotic lineages. For each species, genes have been clustered using the H3K4me3 signal, forming the following four clusters: high, middle, low and no signal. For *N. gruberi*, because the amount of methylated genes is small, the classification was done independently, dark blue corresponds to 6mA-methylated genes, pale blue corresponds to genes with H3K4me3 with no 6mA and orange for genes that lack both H3K4me3 and 6mA. H3K4me3 and H3K27ac are shown as CPM.

Our findings align with prior reports indicating an enrichment of 6mA in nucleosomal linker DNA^16–18,32^. In ciliates, the deletion of AMT1 disrupts 6mA patterns, resulting in more diffuse nucleosome positioning around the TSS^18,28^. Most species in our dataset also exhibit a periodic 6mA pattern. However, *Naegleria*, *Acanthamoeba* or *Chromosphaera* lack a discernible periodic 6mA pattern (Extended Data Fig. 7). The absence of periodicity might be attributed to irregular nucleosome positioning in these species or potential issues with TSS annotations. However, we could not detect periodic patterns over individual genes, unlike in species that display periodicity (Fig. 4b). Moreover, there is a considerable variation in the average number of nucleosomes covered in 6mA territories across species. For instance, the stramenopile *Aurantiochytrium limacinum* displays only three 6mA peaks, while the metamonad *T. vaginalis* exhibits periodic 6mA peaks across entire gene bodies, albeit with decreasing intensity (Fig. 5 and Extended Data Fig. 7). Thus, the 6mA–nucleosome linker association is consistent across AMT1-encoding eukaryotes, although with lineage-specific variations.

**Extended Data Fig. 9:**
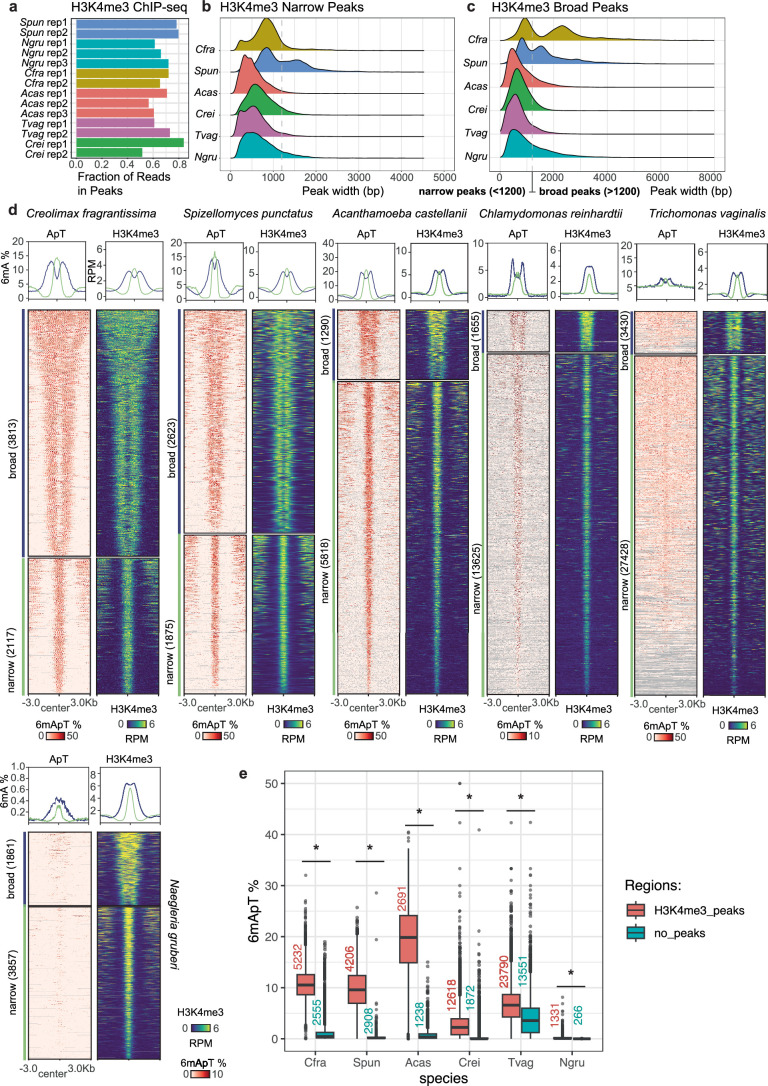
6mA is located within H3K4me3-demarcated regions. **a**, Fraction of reads falling within MACS3 narrow peaks for all H3K4me3 datasets used in this study. **b**, Distribution of peak sizes called as narrow peaks. **c**, Broad peaks, in each species. **d**, Heatmap showing the % of 6mA at ApT sites and H3K4me3 ChIP–seq coverage on both broad (≥1200 bp) and narrow (<1200 bp) H3K4me3 peaks (as defined in **c**, with average metaplots summarizing the data). ChIP–seq data are presented as reads per million (RPM), and each plot is centered on the H3K4me3 peak midpoint. **e**, Boxplot of the percentage of 6mA at ApT sites on H3K4me3 peaks compared to genomic regions that do not overlap H3K4me3 peaks (spanning less than 3500 bp). Boxplots show the median (centerline), interquartile range (box), and the smallest and largest values within 1.5× IQR (whiskers); points outside this range are plotted as outliers. An asterisk indicates a Wilcoxon ranked test (one-sided) p-value < 2.2 × 10^−16^. Cfra, *Creolimax fragrantissima:* Spun*, Spizellomyces punctatus*; Acas, *Acanthamoeba castellanii*; Crei*, Chlamydomonas reinhardtii*; Ngru, *Naegleria gruberi*; Tvag, *Trichomonas vaginalis*. Source data

To probe what shapes 6mA patterns and their link to nucleosomes, we performed chromatin immunoprecipitation sequencing (ChIP–seq) for two TSS-associated and transcriptionally active histone marks—histone 3 lysine 4 trimethylation (H3K4me3) and lysine 27 acetylation (H3K27ac)^28,54^. We generated data for four species and reanalyzed datasets for two additional species^55^, covering most divergent eukaryotic groups with comparable ChIP–seq quality (Extended Data Fig. 9a and Supplementary Fig. 4). Both histone marks showed expected enrichments post-TSS with peak widths under 1,000 bp (Fig. 5 and Extended Data Fig. 9b,c)^28,56^. We then grouped genes by H3K4me3 signal and found that 6mA closely overlaps with this histone mark (Fig. 5 and Extended Data Fig. 9d). In contrast, genomic regions beyond H3K4me3 peaks have substantially lower 6mA levels across all species (Extended Data Fig. 9e). Notably, 6mA aligns with H3K4me3 not only in intensity but also in deposition width.

**Extended Data Fig. 10:**
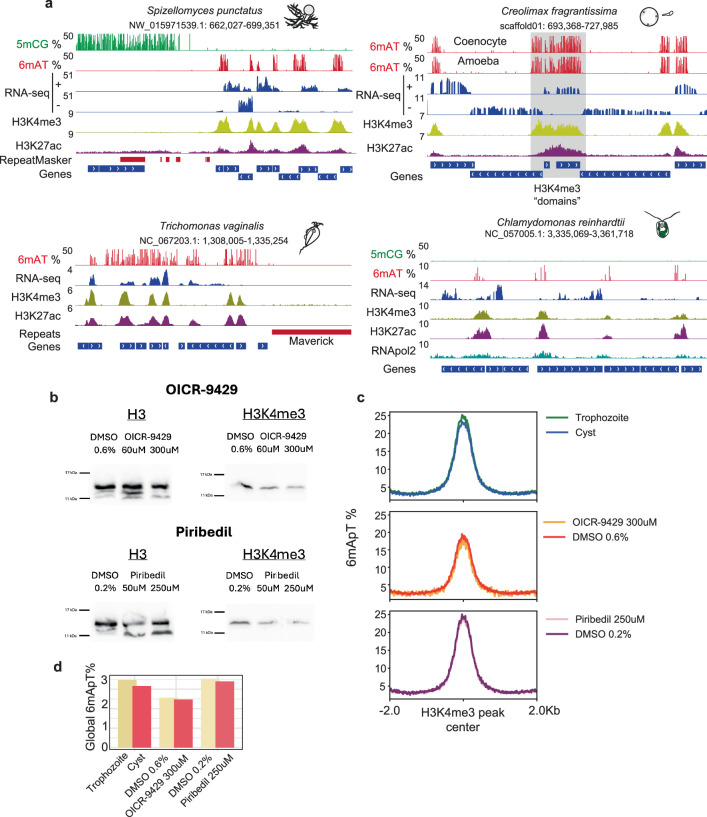
Pharmacological H3K4me3 depletion does not result in lower 6mA. **a**, Genome browser snapshots from four species, with genomic coordinates indicated at the top. RNA-seq (displayed for both positive and negative strands) and ChIP–seq datasets are normalized using reads per million (RPM). Non-stranded RNA-seq data are shown as a single track. For *Chlamydomonas reinhardtii*, RNA polymerase II ChIP–seq data are also displayed. **b**, Western blot analysis showing total histone H3 and H3K4me3 modifications in *A. castellanii* cells treated with the H3K4me3 inhibitors OICR-9429 and Piribedil. Equal amounts of total protein were loaded in each well. This experiment was repeated twice. **c**, Average 6mA at ApT (%) levels measured on H3K4me3 peaks in wild-type cells, comparing OICR-9429 treatment versus control and Piribedil versus control. DMSO concentrations for controls are matched to the final DMSO levels in the inhibitor samples. **d**, Global 6mA at ApT (%) levels in each sample as described in **c**. Source data

In the ichthyosporean *C. fragrantissima*, some genes were fully covered by H3K4me3, extending beyond typical post-TSS patterns (Fig. 5 and Extended Data Fig. 9d). These broad H3K4me3 peaks cannot be explained solely by head-to-head gene arrangements (Extended Data Fig. 9c,d), and have been associated with low transcriptional levels^57^. Within these H3K4me3 domains, 6mA is present throughout (Extended Data Fig. 10a), supporting a tight link between their deposition in *Creolimax*. Interestingly, in ichthyosporeans, the DNA-binding partner of AMT1—the p1 subunit—contains a PHD domain, which is known to read histone tail methylations^58^ (Supplementary Fig. 5).

The other two species displaying divergent 6mA and H3K4me3 patterns are the heterolobosean *N. gruberi* and the metamonad *T. vaginalis*. In *Naegleria*, only 644 genes show 6mA, so we categorized genes based on the presence or absence of 6mA and/or H3K4me3. Genes with 6mA showed the highest H3K4me3 signal (Figs. 4b and 5). However, most genes displayed H3K4me3 without 6mA, suggesting independent deposition (Fig. 5 and Extended Data Fig. 9d). In contrast, H3K27ac was comparable between genes with 6mA and those with only H3K4me3. In *Naegleria*, as in most eukaryotes, the p1 subunit lacks a PHD domain, so colocalization with H3K4me3 would require intermediate proteins.

*T. vaginalis* exhibits an opposite pattern; H3K4me3 and H3K27ac correlate with 6mA intensity, but 6mA extends beyond the modified nucleosomes (Fig. 5 and Extended Data Figs. 9d and 10a). In *Trichomonas*, both 6mA and H3K4me3 may depend on transcriptional initiation, but 6mA extends further, spanning broader domains. *Trichomonas* is exceptional as it lacks p1, p2 and AMT6/7 orthologs that could explain this divergent pattern (Fig. 1c).

To directly test whether the H3K4me3 deposition influences 6mA presence, we treated *A. castellanii* with H3K4me3 inhibitors. We used OICR-9429 and Piribedil, which block WDR5 interactions with histone methyltransferases^59,60^. Both inhibitors resulted in a dose-dependent reduction in H3K4me3 levels while leaving overall H3 levels unaffected (Extended Data Fig. 10b). At the highest inhibitor concentrations, we profiled the 6mA methylome in treated and DMSO control samples. Furthermore, 6mA levels showed minimal differences between inhibitor and control groups, both globally and at H3K4me3 peaks (Extended Data Fig. 10c,d), suggesting that H3K4me3 does not directly determine 6mA deposition in these species.

Overall, our data suggest that AMT1-dependent 6mA is tied to nucleosome positioning across eukaryotes, especially with transcription-permissive marks. The hierarchy between these components appears lineage-specific and may be simplified in some lineages.

## Discussion

**Fig. 6:**
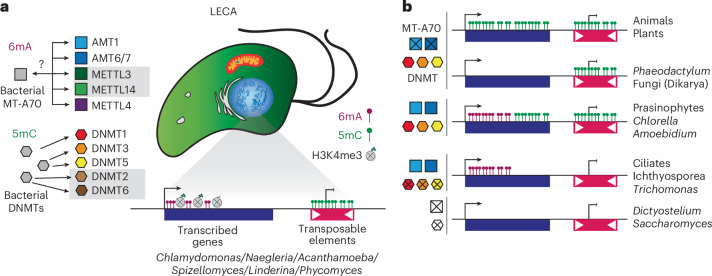
Reconstruction of the LECA epigenome. **a**, Diagram showcasing the distinct evolutionary origins of 6mA (MT-A70) and 5mC (DNMTs) enzymes in the advent of the LECA, and the inferred ancestral epigenome based on distribution and proposed roots of the eukaryotic taxa. Species genus represents examples of extant eukaryotes that still exhibit the inferred ancestral patterns. The question mark symbol indicates that potentially prokaryotes might have acquired MT-A70 sequences from a pre-eukaryotic lineage. **b**, Derived epigenome patterns in modern eukaryotes associated with simplification in methyltransferase repertoires, with example species/lineages for each pattern. Crossed rectangles or hexagons indicate enzyme losses, which are color coded and shaped as in **a**. The presence of DNMTs does not imply co-occurrence of all three enzymes, but any of them.

We show that 6mA is widespread across eukaryotes and trace its origin to the LECA. Our broad sampling supports an ancestral AMT1 pathway, irrespective of the debated root positions of the eukaryotic tree of life^61–63^. Unlike 5mC DNMTs acquired by LECA through independent bacterial transfers^64^, the 6mA MT-A70 methyltransferases were likely inherited in a single event from a bacterial donor (Fig. 6a). Pre-LECA, MT-A70 underwent duplications into six families, undergoing subfunctionalisation in heterodimeric partnerships and RNA or DNA substrates. Postdiversification, MT-A70 families remained relatively static in eukaryotic evolution, contrasting with DNMT families, which underwent lineage-specific duplications and expansions (Dim-2/RID in fungi, CMT and DRM in plants)^3,4,65^. Concurrently, 6mA patterns remained relatively constant across AMT1-encoding eukaryotes, consistently enriching ApT dinucleotides at TSS. Conversely, 5mC is variable, ranging from gene-body methylation to TE silencing, and methylates sequence contexts beyond CpGs^4–6^. As 5mC is presumed to ancestrally silence TEs^4,6,53^, its faster evolutionary dynamics may result from adaptations to track recurrent transposon invasions, in contrast to the slower concerted co-evolution of the 6mA–AMT1 pathway targeting endogenous genes. Similarly, the targeting of silencing histone modifications appears to evolve more dynamically than those of transcriptionally associated modifications^57^. Our survey suggests that LECA had a dual methylation system, with transcriptionally associated 6mA and repressively associated 5mC (Fig. 6a).

Our study confirms Oxford Nanopore as a reliable platform for 6mA detection^33,66^, showcasing robust and reproducible patterns across species with diverse genome compositions. Long-read base-pair resolution maps avoid pitfalls of other methods^24^. As an example, in *T. vaginalis*, antibody-based pull-downs suggested 6mA enrichment in TE^67^, but this genome is dominated by recently duplicated Maverick retrotransposons^68^, where short-read mapping artifacts are common^25,69^. By contrast, our nanopore data show a strong periodic 6mA pattern on transcribed genes in *Trichomonas*, without enrichment in TEs, consistent with patterns across other eukaryotes^16–18^. Nanopore can provide independent confirmation of conflicting PacBio CLR results, as in *C. elegans*, where 6mA presence remains debated^10,20,70,71^. In *C. elegans*, the proposed 6mA methyltransferase METL-9 belongs to a different family than MT-A70, targeting GGAG rather than ApT^70,71^. Alternative 6mA DNA pathways may exist in eukaryotes, such as the fungal MetB asymmetric methyltransferase^15^, acquired through lateral gene transfer from bacteria. However, in nematodes, the few thousand 6mA sites detected are poorly reproducible across samples and lack the clustered patterns of AMT1-encoding species, likely reflecting the absence of a maintenance methyltransferase^71^. Alternatively, nucleotide salvage pathways incorporating m6A from RNA and mitochondrial DNA into nuclear DNA, or RNA:DNA hybrids, might explain contentious previous reports^22–24,72,73^. By contrast, robust and reproducible ApT methylation in AMT1-encoding eukaryotes suggests a stable, functionally important chromatin component.

Our finding of recurrent AMT1 pathway loss challenges assumptions about the evolution of DNA methylation. Similar to DNMTs and the RNA m6A METTL3/METTL14 pathway, the 6mA–AMT1 pathway is frequently lost across eukaryotes^4,45^. As 6mA shows no obvious mutagenic effect, these losses may reflect evolutionary contingency, with 6mA role compensated by alternative mechanisms. This has produced diverse combinations of 6mA and 5mC across extant eukaryotes (Fig. 6b).

Thus, 6mA strongly correlates with transcription, is enriched with highly expressed genes, yet its dynamics appear limited. Across five species in our dataset, 6mA shows little change under transcriptional perturbations. In another recently published example, the fungi *Phycomyces blakesleeanus* and *Mucor lusitanicus* also displayed modest 6mA dynamics despite treatment-specific transcriptional changes^15^. Similarly, AMT1 knockouts in ciliates and *M. lusitanicus* result in limited transcriptional changes^18,74^. This resembles gene-body 5mC in plants and invertebrates, stably expressed genes are usually methylated, yet developmental transcriptional changes rarely alter 5mC patterns^4,75,76^. Likewise, while H3K4me3 is closely associated with transcription, its direct role in initiating transcription remains debated^77^. For instance, depleting H3K4me3 can increase RNA polymerase II pausing without affecting transcriptional initiation^78^. Overall, chromatin features such as 6mA, gene-body 5mC and H3K4me3 mark transcriptionally active regions but likely act in permissive or downstream roles rather than directly regulating transcription.

The function of 6mA in nucleosome positioning remains unclear, as ciliates with mutated AMT1 exhibit disordered nucleosomes yet remain generally viable^18,28^, primarily only lethally affected in sexual reproduction. In ciliates, the effect on nucleosome positioning is also stronger in vitro than in vivo^18,28,32^. Some of our surveyed species lack a periodic 6mA pattern, questioning the importance of 6mA in nucleosome positioning. In contrast, our data suggest an ancient link between 6mA and H3K4me3, perhaps also with H2A.Z^28,74^. Given the widespread presence of H3K4me3 as a post-TSS marker^54^, even in species lacking 6mA, the latter may be nonessential or readily substituted in this region. Notably, H3K4me3 and H2A.Z usually anticorrelate with 5mC across eukaryotes^53,79,80^, so the H3K4me3–6mA link may further contribute to chromatin compartmentalization. Our inhibitor experiments in *A. castellanii* show that H3K4me3 is not required for 6mA deposition. In ichthyosporeans, the p1 PHD domain suggests possible H3K4me3 recognition. Thus, the hierarchical relationship between H3K4me3–6mA may have evolved independently, or their co-occurrence may simply reflect a downstream consequence of transcription rather than a direct causal link. Biophysically, 6mA is believed to decrease double-strand DNA stability, an effect potentially advantageous for transcription at the start of highly expressed genes^22,27^. Yet to understand the 6mA role as an epigenetic mark, identifying potential readers is key. Similarly, although 5mC stiffens DNA^22^, its function across eukaryotes depends more on associated readers or inhibiting transcription factor binding than on intrinsic biophysical properties^81^.

Intriguingly, 5mC is predominantly retained in major multicellular eukaryotic lineages (plants, animals and Dikarya fungi), whereas the AMT1–6mA pathway was lost in these lineages. In plants and animals, 5mC evolved to methylate gene bodies (Fig. 6b)^4,5,53^, replacing 6mA in the post-TSS region, yet this substitution is unlikely to be the sole cause of 6mA loss. The unicellular relatives of plants and animals have gene-body 5mC coexisting with 6mA, suggesting 6mA and 5mC have distinct roles even if found in the same regions (Fig. 6b). In conclusion, our data reshape the understanding of 6mA function and evolution, highlighting that both 6mA and 5mC were integral to the original eukaryotic chromatin toolkit. Although ciliates have spearheaded our understanding of 6mA in eukaryotes, their drastically unusual genome organization, with a micronucleus and a macronucleus, and a lineage-specific expansion of MT-A70s^18,28^, will benefit from complementary insights from alternative lineages. By expanding taxon sampling to tractable species with canonical genomes, our work sets the stage to clarify 6mA functions and to understand why this pathway was lost in major multicellular lineages.

## Methods

Experiments within this study did not require ethics board approval.

### Sequence search and phylogenetic analysis

A collection of eukaryotic proteomes spanning the broadest diversity of lineages was scanned using HMMER3 (v3.3.1) with the PFAM domain for MT-A70 (ref. ^83^), using an *e* value of 0.0001 as a threshold. Additionally, AMT6/7 sequence from *Tetrahymena termophila* was used as a query against the same database using BLASTP (2.9.0+), to obtain divergent orthologs that were filtered out with the hmmsearch approach. In parallel, *T. termophila* MT-A70 AMT1 and AMT7 sequences were used to BLASTP against NCBI ‘nr’ databases, excluding eukaryotes as taxonomic hits. The resulting proteins were merged into a multisequence alignment using MAFFT L-INS-i mode^84^ (v7.475). The alignment was trimmed with trimAl -gappyout parameter^85^ (v1.4.rev15), and a maximum-likelihood phylogeny was obtained using IQ-TREE^86^ (v2.1.2), with automatic model selection, computing 1,000 ultrafast bootstraps and ALRT replicates as nodal supports. The distribution of MT-A70 hits across our dataset can be found in Supplementary Table 1. To determine the distribution of MT-A70 in prokaryotes, the same BLASTP approach was used, allowing 250 hits against the NCBI ‘refseq_select’ database, specifying bacteria and archaea (separately). The 302 Asgardarchaeota genomes were downloaded from NCBI (January 2025) and searched using HMMER3, as for eukaryotes. Another phylogeny was constructed from a subset of the original tree, selecting a few representative eukaryotic species and all prokaryotic outgroups, using the same methods as for the larger tree. To determine the presence of p1 and p2 orthologs, we used BLASTP against the custom eukaryotic proteome database, requiring an *e* value of <0.0001.

### Protist cultures

*C. fragrantissima, Abeoforma whisleri* and *C. limacisporum* cells were grown axenically in marine broth liquid medium (Difco, 2216) at 17 °C, 17 °C and 23 °C, respectively. *C. perkinsii* cells were grown axenically at 23 °C in liquid medium (containing 3 g of yeast extract, 3 g of malt extract, 5 g of peptone, 10 g of glucose and 20 g on NaCl per liter of distilled water). *A. castellanii* Neff cells were grown axenically at 23 °C in ATCC medium 712. *N. gruberi* cells were grown axenically at 23 °C and 30 °C in ATCC medium 1034. *A. limacinum* cells were grown axenically at 19 °C in ATCC medium 790. *S. punctatus* cells were grown axenically at 17 °C in liquid medium (containing 2.5 g of yeast extract, 0.5 g of K_2_HPO_4_, 2.5 ml of ethanol, 15 ml of glycerol and 485 ml of Milli-Q water). *D. discoideum* was grown axenically at 23 °C in liquid HL5-C medium (Formedium). *C. reinhardtii* CC-503 was grown on TAP growth media (Thermo Fisher Scientific, A1379801) at room temperature.

We performed DNA extractions for *N. gruberi*, *D. discoideum*, *C. reindhardtii*, *C. perkinsii*, *A. whisleri* and *S. punctatus* from confluent cultures grown into 25-ml flasks for 7 days, and 4 days for *C. limacisporum*. For *A.limacinum*, cells were grown for 7 days and then 1 ml was passed into two 25-ml flasks with fresh medium to enrich for zoospores and then collected 2 days later. In all cases, cells were centrifuged at 3,000*g* for 5 min, and the supernatant was discarded before DNA extraction.

### Stage-specific sampling

For *A. castellani* trophozoite DNA, we obtained cells from a confluent 7-day culture. For the cystic stage, cells were grown for 5 days in 25-ml flasks and then media was removed and replaced by 5 ml of encystment medium (containing 3.728 g of KCl, 1.68-g NaHCO_3_, 0.986 g of MgSO_4 _× 7H_2_O, 0.03 g of CaCl_2 _× 2H_2_0 and 0.017 g of 2-amino-2-methyl-1,3-propanediol per 500 ml of distilled water)^87^; after 3 days of incubation at 23 °C, *A. castellani* cysts were collected for DNA extraction.

*C. fragrantissima* cells were grown until confluency for 5 days in 25-ml flask, and then cells were scratched and passed into 250-ml flask with 25 ml of fresh medium. These new flasks were grown for 48 h under gentle agitation at 17 °C and then were filtered using a 20-μm cell strainer (pluriSelect) and collected into a 50-ml Falcon tube to separate the amebas from the mature coenocytes, as described previously^41^. Both amebas and coenocytes were collected for independent DNA extraction.

For *C. perkinsii* synchronization, we filtered a 6-day-old culture using a 5-µm filter (puriSelect) to obtain a small-celled population that was then diluted 1:100 in liquid media to grow at 23°C, as described before^42^. Cells were visually inspected and collected after 54 h and 96 h, and then snap-frozen in liquid N_2_ for later DNA extractions.

For *S. punctatus*, zoospore cells were grown until confluency for 7 days in 25-ml flask, and then 1 ml of the culture was spread onto *Spicellomyces*-agar plates (*S. punctatus* liquid medium + 15 g l^−1^ of agar) and incubated for 48 h at 30 °C. After that, the active *Spizellomyces* plates were flooded with 1 ml of dilute salt (DS) solution and incubated at room temperature for 1 h to induce the release of zoospores. After the release, the DS solution containing the zoospores was retrieved and filtered using a 10-μm syringe strainer (pluriSelect) as described previously^43^. At that moment, zoospores were collected and frozen in liquid N_2_ for later DNA and RNA extractions. For the multinucleated colonies, zoospores obtained as described before grown in liquid media for 24 h at 30 °C, multinucleated cells were collected by scraping the flask and snap-frozen in liquid N_2_ for later DNA and RNA extractions.

### Nucleic acid isolation

Before DNA extraction, to break the cell wall of *S. punctatus*, *C. fragrantisiima* and synchronized *C. perkinsii* samples, heat shock was applied, the cells were frozen with liquid N_2_ and then thawed at 60 °C, repeating this thrice. For *C. perkinsii* unsynchronized samples and *A. castellanii* cysts, we immersed the pellets in liquid N_2_ and then ground them with a pestle and mortar.

The resulting cell pellets for *N. gruberi, A. castellanii* amebas, cysts and Piribedil-treated samples*, D. discoideum*, *C. perkinsii* unsynchronized samples, *C. limacisporum* and *A. limacinum* were used for DNA extraction using the Qiagen MagAttract HMW DNA kit following the manufacturer’s whole blood protocol. For *S. punctatus*, *C. fragrantissima, C. perkinsii* synchronized samples*, A. castellanii* OICR-9429 treated samples and *C. reindhartii* NEB Monarch Genomic DNA Purification Kit were used following the animal tissue protocol. For *A. whisleri*, we used a phenol/chloroform genomic DNA extraction method.

Total RNA was extracted in biological duplicates from *N. gruberi* cultures grown at 23 °C and 30 °C, as well as from *S. punctatus* zoospores and multinucleate colonies, following NEB Monarch Total RNA Miniprep Kit tough-to-lyse samples protocol after snap-freezing the samples in liquid N_2_.

The genomic DNA of *T. vaginalis* strain G3 was obtained from ATCC, and the DNA of *M. pusilla* Culture Collection of Algae and Protozoa (CCAP) 1965/4, *Chlorella variabilis* NC64A and *Cyanophora* *paradoxa* CCAP 981/1 were obtained from CCAP (Oban).

### Nanopore sequencing, base calling and methylation analysis

We quantified genomic DNA using a Qubit 3 Fluorometer with the dsDNA BR Assay Kit and assessed DNA size with a TapeStation 2200 using the Genomic DNA ScreenTape Assay. We then started with 1−1.5 μg of high molecular weight genomic DNA that was ligated with nanopore SQK-LSK110 ligation kit following the manufacturer’s instructions and sequenced later in MinION R9.4.1 flow cells (see Supplementary Table 3 for details).

The resulting fast5 files were then used as input for Guppy (v6.5.7), and the Rerio modified base-calling model ‘res_dna_r941_min_modbases-all-context_v001’ was specified, aligning the reads to the reference genome (--align_ref) parameter. The list of reference genomes is provided in Supplementary Table 4 (refs. ^41,45,88–91^). The resulting BAM files were then merged and sorted using samtools, and then the Oxford Nanopore ‘modbam2bed’ (v0.9.1) was used to obtain either 6mA (-m 6mA) or 5mC (-m 5mC) basecalls. The resulting bedMethyl files were then used to extract the neighboring bases for each A position, gathering one base upstream and two downstreams using BEDTools^92^, and the stranded (5′ to 3′) four-nucleotide context was included for each position. Dinucleotide contexts were divided into AT, AA, AC and AG, and bigwig files were generated using the UCSC bedGraphToBigWig tool (v4). Visualization of average methylation levels on genes, TSS and TEs was obtained using DeepTools2 (v3.5.0)^93^.

For *N. gruberi* temperature-treated DNA samples, we generated libraries using the SQK-LSK114 ligation kit and sequenced them using the PromethION R10.4.1 flow cells. Because there is no compatible R10.4.1 model for Guppy, we switched to Dorado (v0.7.2) for 6mA base calling, using 6mA in sup mode. The 6mA calls were obtained using the Oxford Nanopore Modkit (v0.4.2) pileup function, testing several thresholds for calling a modified base, inspecting them in the browser and finding that 0.995 showed the lowest false positive rate when compared to R9.4.1 for *N. gruberi* (--motif AN 0 --mod-thresholds a:0.995).

Global methylation levels were computed in R, and the regional average levels of 6mA were computed using the Bioconductor bsseq package^94^, treating the ApT dinucleotides as if they were CpGs. To call an ApT site methylated, we required at least 10× coverage and >10% 6mA, with lower coverage thresholds in *M. pusilla* and *A. whisleri* (5×) and in *C. limacisporum* and *C. variabilis* (3×). To call methylated genes, we required that each gene contain at least three methylated ApT sites. To compute stage-specific methylation differences in gene bodies, we computed the weighted average of 6mApT methylation across the whole gene bodies and subtracted those values. Genes with a difference of >1.5% 6mApT in both directions were selected as the most highly distinct.

### Selection analysis

To assess selective pressure acting on AMT1 gene sequences from the species sequenced in this study, a maximum-likelihood phylogenetic tree was inferred using IQ-TREE, as described in the phylogenetic methods section. A codon-based alignment was generated by combining the protein sequence alignment obtained with MAFFT with the corresponding nucleotide sequences using PAL2NAL (v14). Tests for positive selection were performed using the CodeML implemented in EasyCodeML software (v1.41)^95^, applying the preset Site Model to detect the variation in selective pressure across codon sites.

### RNA-seq preparation and analysis

We used 0.8–1 μg of total RNA from *N. gruberi* cultures grown at 23 °C and 30 °C and *S. punctatu*s cell stages to select poly-A tails with the NEBNext Poly(A) mRNA Magnetic Isolation Module kit; then libraries were prepared using the NEBNext Ultra II Directional RNA Library Prep Kit for Illumina in duplicate, following the manufacturer’s instructions. The resulting libraries were sequenced on a NovaSeq 2000 (NovoGene), yielding 20–30 million paired-end reads per replicate.

Furthermore, publicly available RNA-seq datasets for all species were downloaded from ENA from various studies (Supplementary Table 5)^40–42^. The data were mapped to the reference genomes using HISAT2 (v2.2.1)^96^, restricting intron size to 40,000 bp. To calculate steady-state transcription levels across all species, StringTie was used to obtain the Transcripts per Million measure for all gene models, and bigwig files were generated using DeepTools2, specifying the stranded information when the original libraries had strand-specific information^97^. For the differential expression analysis, Kallisto (0.46) was used to obtain the counts (est_counts) by aligning reads to reference transcriptomes^98^. The estimated counts were read into R and DESeq2 was used to compute the differential expression, using a *P*-adjusted value of <0.01 as a significant threshold.

### ChIP–seq library preparation and analysis

ChIP–seq was performed as previously described^99^ with modifications. Briefly, 100-ng chromatin per species was pooled per ChIP. Pooled chromatin was incubated at 4 °C for 12–14 h with rotation with 2.5-µg anti-H3K27ac (Abcam, ab4729) or 2.5-µg anti-H3K4me3 (Sigma-Aldrich, 07-473). Immunoprecipitated complexes were captured using a mix of Protein A (Sigma-Aldrich, 16-661) and Protein G magnetic beads (Sigma-Aldrich, 16-662), washed and reverse crosslinked for 30 min at 55 °C followed by an hour at 68 °C. Immunoprecipitated DNA was purified using solid-phase reversible immobilization beads (Beckman Coulter, A63881). The NEBNext Ultra II DNA Library Prep Kit (New England BioLabs) was used to produce the resulting ChIP libraries according to the manufacturer’s protocol.

The data for *C. reinhardtii* and *T. vaginalis* were downloaded from ENA, belonging to previous studies^55,100^ (Supplementary Table 6, SRR numbers).

All ChIP–seq data were analyzed using fastp to trim reads, and the reads were mapped to the genomes using bowtie2 (v1.2), allowing a maximum insert size of 2,000 bp (-I 2000). Duplicate reads were removed using Sambamba (v0.6.6). DeepTools2 was used to generate bigwig files, compute insert size distributions and visualize epigenomic data, as well as the Integrative Genome Viewer. MACS3 (v3.0.0a7) was used to call peaks^101^, using a *q* value of 0.01 and adjusting the genome size to each species (for example, -q 0.01 -g 4e7), as well as activating the broad parameter for broad peak calling. Broad peaks were defined as those having >1,200 bp using their width distribution as a reference (Extended Data Fig. 9c). The H3K4me3 signal was clustered using DeepTools2 plotHeatmap function, allowing four *k*-means clusters (--k-means 4), providing the four categories shown in Fig. 5.

### Inhibitors and western blot

Histone H3K4me3 suppression drugs OICR-9424 (MedChemExpress, HY-16993) and Piribedil (Cayman Chemical, 3605-01-4) were diluted in DMSO at 50 mM and 100 mM, respectively, as stock solutions. For drug treatment experiments, 2.1 million *A. castellanii* cells were cultured in 42 ml of liquid media at 23 °C for 24 h. After incubation, the cells were treated for 30 h with either 60-µM OICR-9424 or 300-µM OICR-9424, or with 5-µM Piribedil or 250-µM Piribedil. Two control samples were prepared by treating cells with 0.6% DMSO (for OICR-9424) and 0.2% DMSO (for Piribedil), adjusting the DMSO concentration to the highest inhibitor concentrations. Cells were collected by scraping the flasks and centrifuged at 3,000*g* for 5 min, the supernatant was discarded and the pellets were snap-frozen in liquid N_2_ for subsequent histone and DNA extraction.

Histones were extracted using an acid extraction protocol optimized for western blot analysis. Briefly, treated or control *A. castellanii* pellets were washed twice with ice-cold PBS. Cells were resuspended in Triton Extraction Buffer (TEB; PBS containing 0.5% (vol/vol) Triton X-100, 5 mM sodium butyrate and one Complete Mini EDTA-free protease inhibitor tablet per 10 ml) at a density of 3 × 10^−^⁷ cells per ml. Cell lysis was performed on ice for 10 min with gentle agitation. Nuclei were pelleted by centrifugation at 650*g* for 10 min at 4 °C. The nuclei were washed once with half the volume of TEB and centrifuged again under the same conditions. The resulting nuclear pellet was resuspended in 0.2 N HCl at a density of 12 × 10⁷ nuclei per ml and incubated overnight at 4 °C to acid-extract histones. Samples were then centrifuged at 650*g* for 10 min at 4 °C to remove debris. The supernatant containing soluble histones was collected and neutralized with 2 M NaOH at one-tenth of the volume of the supernatant. Histone extracts were aliquoted and stored at −20 °C until further use.

Protein samples (5 µg for H3 and 12 µg for H3K4me3 detection) were mixed with 2× Laemmli sample buffer containing 50-mM dithiothreitol and denatured by boiling at 100 °C for 10 min. Proteins were separated by SDS–PAGE on 15% acrylamide gels and transferred to nitrocellulose membranes. Membranes were blocked for 1 h at room temperature in TBST buffer (50 mM Tris–HCl, pH 7.5; 150 mM NaCl; 0.05% (vol/vol) Tween-20) containing 5% (wt/vol) nonfat dry milk.

Primary antibodies were incubated for 2 h at room temperature in TBST containing 1% (wt/vol) nonfat dry milk. After washing, membranes were incubated with horseradish peroxidase-conjugated secondary antibodies diluted 1:10,000 in TBST with 1% (wt/vol) nonfat dry milk.

Signal of the secondary antibody was detected with a FUSION Solo S (Vilber Lourmat).

The primary antibodies used were antihistone H3 (1:1,000; Abcam, ab1791), histone H3K4me3 (1:500; Active Motif, AB_2615077) and the secondary antibody goat antirabbit IgG (H + L; Invitrogen, 31462).

### Genome assembly and re-annotation

The genomes of *N. gruberi* and *C. perkinsii* were re-assembled for this study. We used Oxford Nanopore reads and performed conventional base calling with Guppy using the ‘sup’ model and splitting chimeric reads. The resulting reads were then used as input for Flye (v2.9-b1768) using –nanopore_hq option, and allowing two steps of polishing^102^. Publicly available Illumina reads^90^ were used for polishing using HyPo^103^ (v1.0.3). For *N. gruberi*, the resulting contigs were scaffolded using *Naegleria lovaniensis* chromosome-scale genome^104^ with RagTag^105^ (v2.1.0). Publicly available RNA-seq for each species^90,106^ was aligned to the genomes using HISAT2 with the -dta parameter with a maximum intron size of 10 kb, and Stringtie (v2.1.2) was then used to predict transcript models^96,97^. In parallel, Trinity (v2.8.5) was used to build de novo transcriptome assemblies^107^, which were mapped to the genome using GMAP (14 October 2020). The best transcripts were selected using Mikado^108^ (v2.3.0), which were then used to generate exon hints for Augustus^109^ (v3.4.0). Liftoff^110^ (v1.6.3) was used to transfer annotations from the previous genome assemblies to the new assemblies^90,111^, which were then used as coding sequence hints. Then Augustus was trained on the best Mikado gene models for each species, and those species models were used to predict the genes with both exon and coding-sequence hints. In the last step, transcript UTRs were added to the Augustus gene models using PASA (v2.0.1) with the best Mikado transcripts as input^112^.

For *C. limacisporum*, *C. variabilis*, *C. fragrantissima* and *A. whisleri*, the annotations were also updated with available RNA-seq data to include UTRs and improve TSS annotation^41,82,113^. We used a combination of Trinity and Stringtie as described above to generate a nonredundant transcript set with Mikado, which was then used to update gene structures with PASA.

Repeat annotations were generated for species that lacked them, using RepeatModeler2 (v2.0.2a)^114^, including the LTR finding module, and mapping the consensus repeat library against the genome using RepeatMasker (v4.1.2-p1).

### Statistics and reproducibility

No statistical method was used to predetermine sample size. Bulk RNA-seq experiments were performed in two replicates for *N. gruberi* and *S. punctatus*, while all available replicates from public datasets were used for other samples. ChIP–seq experiments were performed with at least two replicates, and Oxford Nanopore sequencing was performed with one replicate per stage or species. Drug treatment experiments and western blot were performed twice with similar results. No data were excluded from the analyses. The experiments were not randomized. The investigators were not blinded to allocation during the experiments or during outcome assessment.

### Reporting summary

Further information on research design is available in the Nature Portfolio Reporting Summary linked to this article.

## Online content

Any methods, additional references, Nature Portfolio reporting summaries, source data, extended data, supplementary information, acknowledgements, peer review information; details of author contributions and competing interests; and statements of data and code availability are available at 10.1038/s41588-025-02409-6.

## Supplementary information

Supplementary Information Supplementary Figs. 1–5.

Reporting Summary

Supplementary Tables 1–6 Supplementary Table 1: CODEML output table highlighting conservation across the AMT1 codon alignment. Supplementary Table 2: Distribution of MT-A70 methyltransferases across the eukaryotic dataset. Supplementary Table 3: Description of Oxford Nanopore libraries generated or used in this study. Supplementary Table 4: Description of the genome assemblies and annotations used for mapping nanopore base modification data. Supplementary Table 5: Description of the RNA-seq datasets used or generated in this study. Supplementary Table 6: Description of the ChIP–seq datasets used or generated in this study.

## Source data

Source Data Fig. 1 Phylogenetic tree of AMT-A70 family in Nexus format.

Source Data Fig. 2 Raw values for Fig. 2a,c,d.

Source Data Fig. 3 Raw values for Fig. 3b–d.

Source Data Extended Data Fig. 1 Phylogenetic tree of AMT-A70 family in Nexus format.

Source Data Extended Data Fig. 2 Raw values for Extended Data Fig. 2a.

Source Data Extended Data Fig. 3 Raw values for Extended Data Fig. 3a,b.

Source Data Extended Data Fig. 4 Raw values.

Source Data Extended Data Fig. 5 Raw values for Extended Data Fig. 5b,d.

Source Data Extended Data Fig. 6 Raw values for Extended Data Fig. 6a,b.

Source Data Extended Data Fig. 9 Raw values for Extended Data Fig. 9a–c,e.

Source Data Extended Data Fig. 10 Raw values for Extended Data Fig. 10d.

Source Data Extended Data Fig. 10 Unprocessed western blots for Extended Data Fig. 10b.

## Data Availability

Nanopore raw sequencing data have been uploaded to BioStudies (https://www.ebi.ac.uk/biostudies/) under the accession S-BSST1363 (ref. ^115^), and the ChIP–seq data can be found in the GEO submission GSE261870. The ChIP–seq for *C. reinhardtii* was obtained from GSE59629, and *T. vaginalis* from GSE89662. Publicly available RNA-seq was obtained from PRJNA794325 (*A. castellanii*), PRJNA360056 (*A. whisleri*), SAMD00394225 (*A. limacinum*), PRJNA1091032 (*C. perkinsii*), PRJNA285347 (*C. reinhardtii*), PRJNA849385 (*T. vaginalis*), PRJNA262632 (*C. limacisporum*), PRJNA210187 (*C. variabilis*), GSE46692 (*M. pusilla*), GSE155535 (*O. tauri*), PRJNA642022 (*N. fowleri*), GSE249241 (*A. appalachense*) and GSE68616 (*C. fragrantissima*). Annotation and other analysis files associated with this article can be found at: 10.5281/zenodo.17174913 (ref. ^116^). IGV genome browser session can be accessed for *C. fragrantissima* (https://tinyurl.com/22zs884x), *S. punctatus* (https://tinyurl.com/2d7jgzpz), *T. vaginalis* (https://tinyurl.com/2arl7o56), *A. castellanii* (https://tinyurl.com/22wjj72j), *N. gruberi* (https://tinyurl.com/mr44r47h) and *C. reinhardtii* (https://tinyurl.com/25aww7qk). Source data are provided with this paper.

## Code availability

All the custom code used for the analysis of this paper can be found on GitHub at https://github.com/AlexdeMendoza/6mA_evolution and Zenodo (ref. ^116^).
