# Supplementary material for: Adenine DNA methylation associated with transcriptionally permissive chromatin is widespread across eukaryotes
Source: Nat Genet. 2025 Nov 18;57(12):3126–36. doi: 10.1038/s41588-025-02409-6 (PMC12695648; doi:10.1038/s41588-025-02409-6)
Supplement: Supplementary file 1 — Supplementary Figs. 1–5. [file 41588_2025_2409_MOESM1_ESM.pdf]

nature genetics

Supplementary information

https://doi.org/10.1038/s41588-025-02409-6

Adenine DNA methylation associated with transcriptionally permissive chromatin is widespread across eukaryotes

In the format provided by the authors and unedited

Supplementary Information for:

Adenine DNA methylation associated with transcriptionally permissive chromatin is widespread across eukaryotes

Pedro Romero Charria, Cristina Navarrete, Vladimir Ovchinnikov, Lan Xu, Luke A Sarre,Victoria Shabardina, Ewa Ksiezopolska, Elena Casacuberta, David Lara-Astiaso, Arnau Sebé-Pedrós, Alex de Mendoza

This file includes:
Supplementary Figures 1 to 5
Captions for supplementary Tables 1 to 6

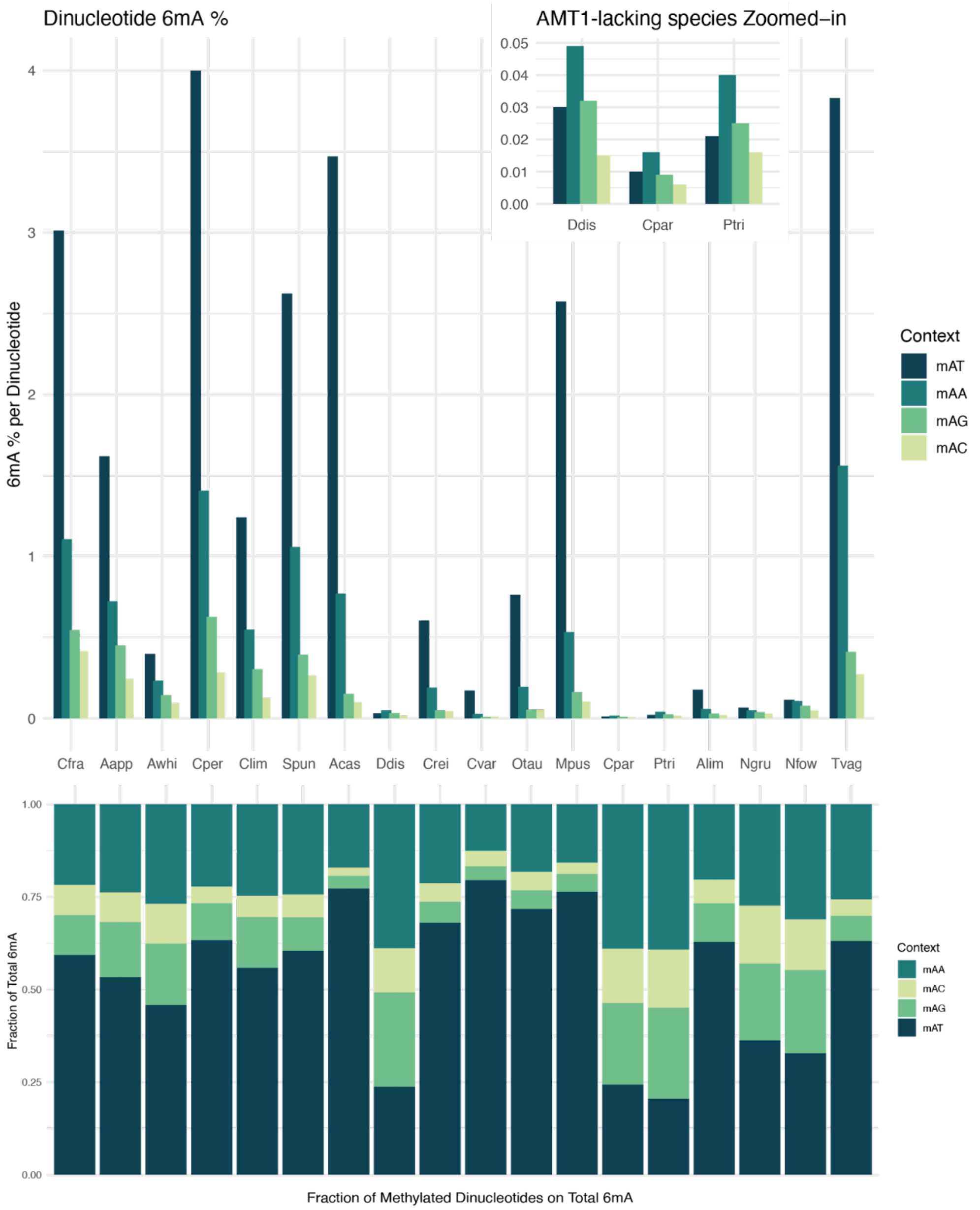

Supplementary Figure 1. Global dinucleotide 6mA shown as total % per species and as fraction of total number of 6mA calls, with Ddis (*Dictyostelium discoideum*), Cpar (*Cyanophora paradoxa*) and Ptri (*Phaeodactylum tricornutum*) as AMT1-lacking species zoomed in. *Cfra - Creolimax fragrantissima, Aapp - Amoebidium appalachense, Awhi - Abeoforma whisleri, Cper - Chromosphaera perkinsii, Clim - Corallocytrium limacisporum, Spun - Spizellomyces punctatus, Acas - Acanthamoeba castellanii, Ddis, Dictyostelium discoideum, Crei - Chlamydomonas reinhardtii, Cvar - Chlorella variabilis, Otau - Ostreococcus tauri, Mpus - Micromonas pusilla, Cpar - Cyanophora paradoxa, Alim - Aurantiochytriumlimacinum, Ngru - Naegleria gruberi, Nfow - Naegleria fowleri, Tvag - Trichomonas vaginalis*.

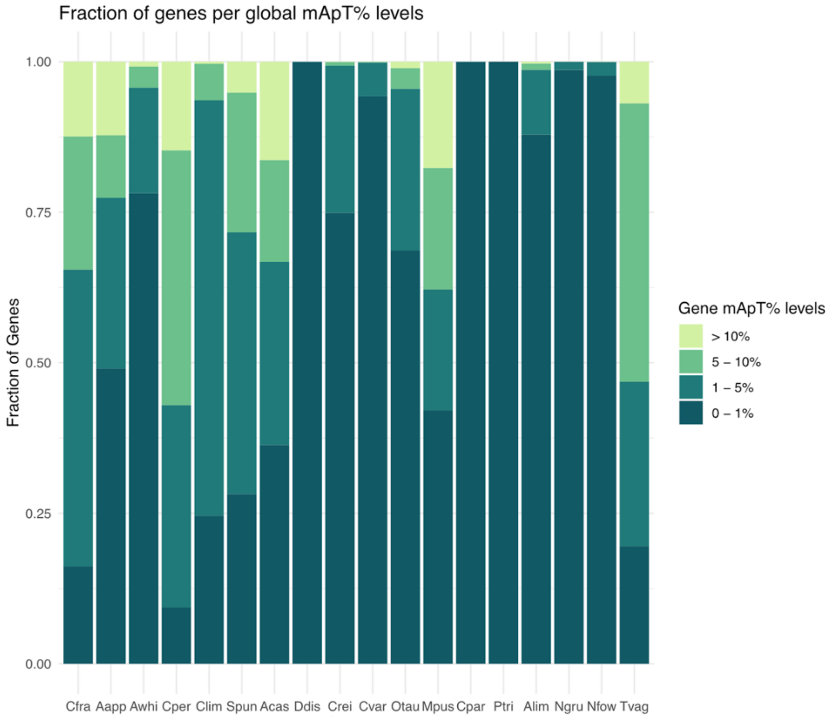

Supplementary Figure 2. Proportion of genes in each 6mApT methylation categories, calculated as the weighted average of 6mApT for the full length of the gene body. Ddis, Cvar and Ptri lack AMT1.

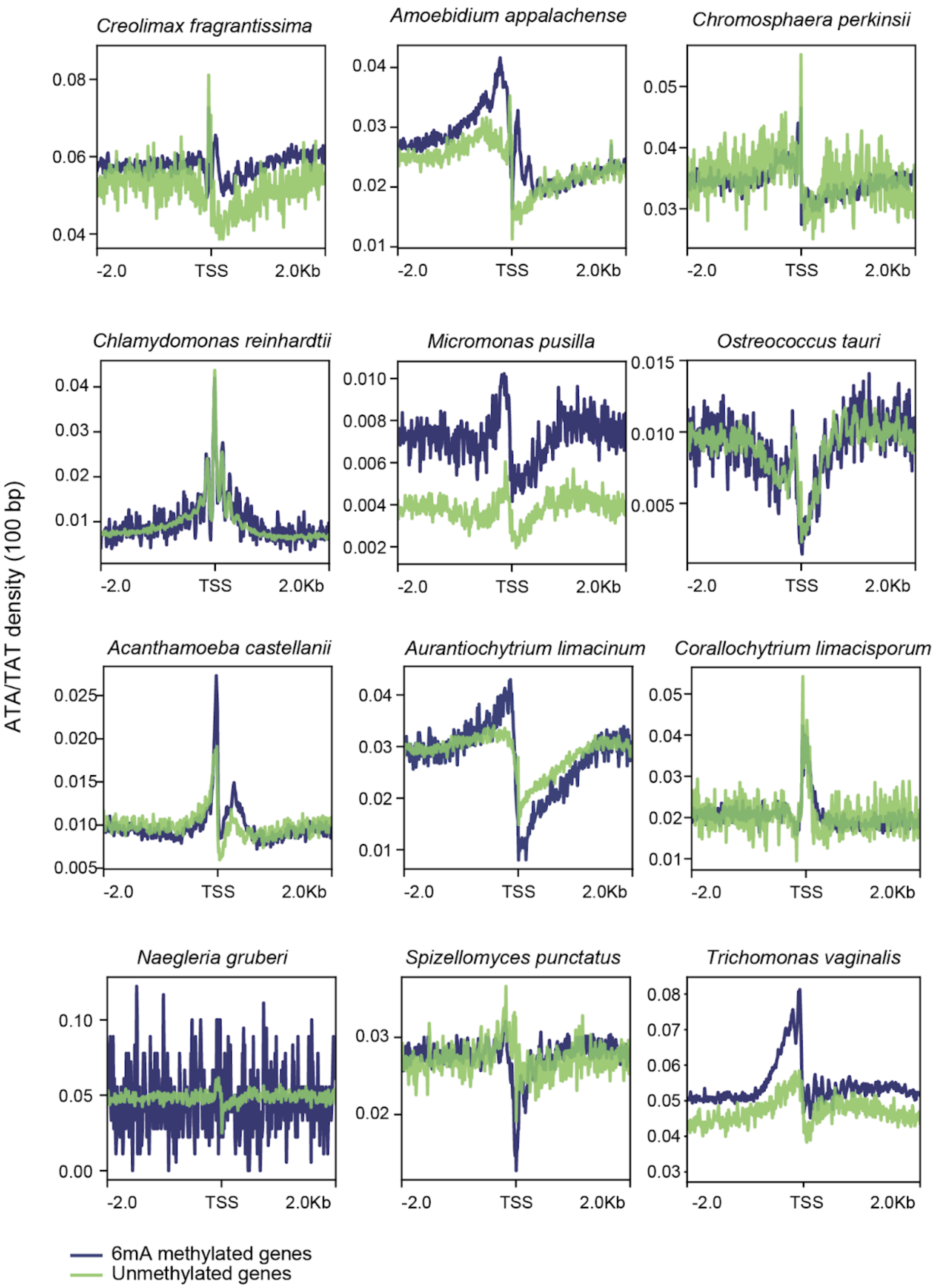

Supplementary Figure 3. Average density of ATA/TAT trinucleotides in 6mA methylated and unmethylated genes, calculated as number of trinucleotides per 100 bp windows.

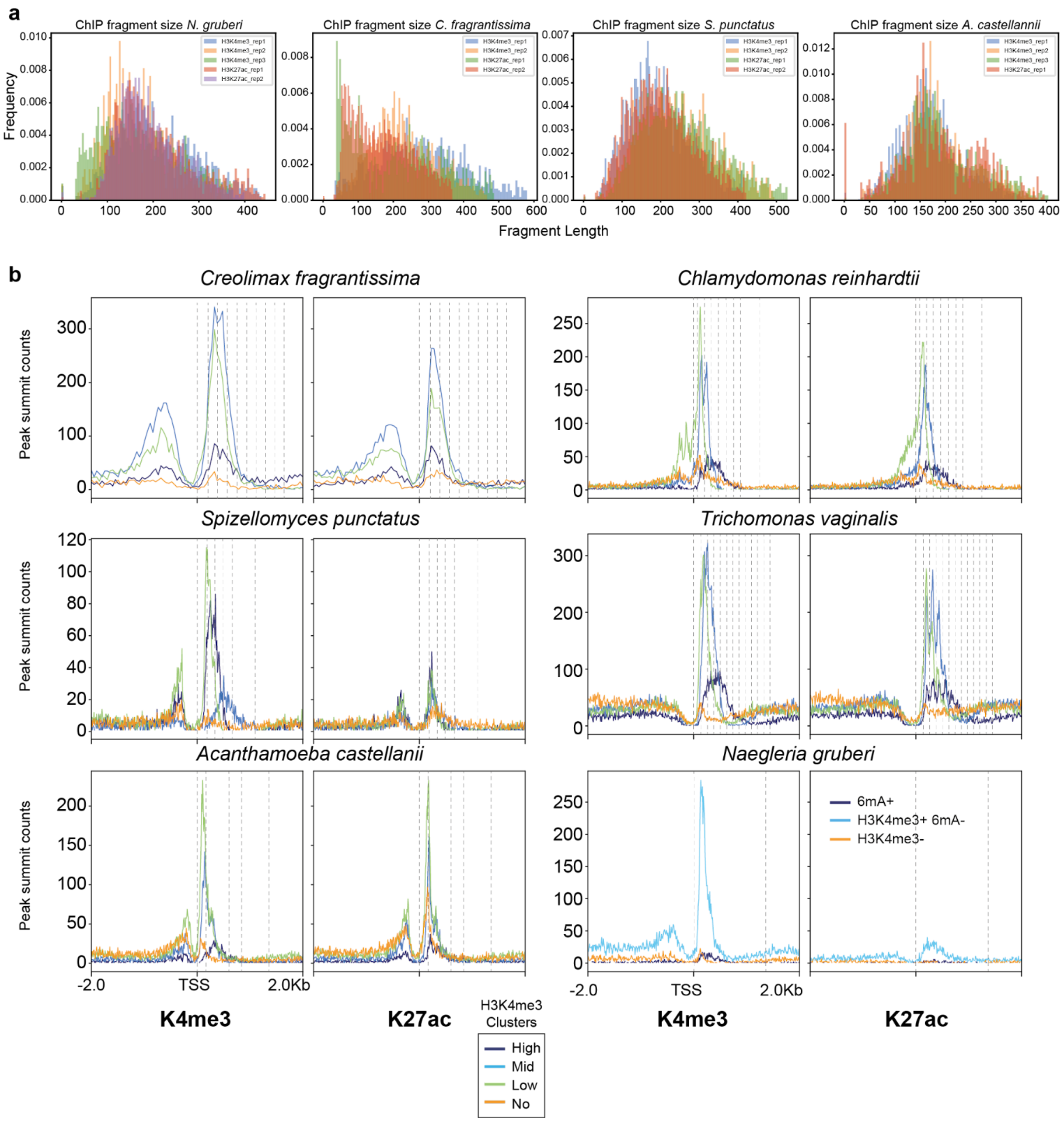

Supplementary Figure 4. (**a**) ChIP-seq insert size distribution for 4 species profiled in this study, shown as per each replicate. (**b**) Distribution of ChIP-seq summits around TSS classified as per H3K4m3 signal, as in Figure 5.

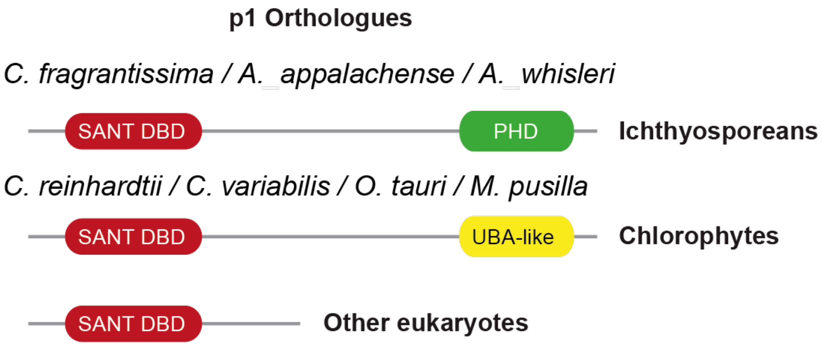

Supplementary Figure 5. Domain architecture of p1 orthologues across the studied species.

Supplementary Table S1. CODEML output table highlighting conservation across the AMT1 codon-alignment.

Supplementary Table S2. Distribution of MT-A70 methyltransferases across the eukaryotic dataset.

Supplementary Table S3. Description of Oxford Nanopore libraries generated or used in this study.

Supplementary Table S4. Description of the genome assemblies and annotations used for mapping Nanopore base modification data.

Supplementary Table S5. Description of the RNA-seq datasets used or generated in this study.

Supplementary Table S6. Description of the ChIP-seq datasets used or generated in this study.
