# Supplementary material for: Adenine DNA methylation associated with transcriptionally permissive chromatin is widespread across eukaryotes
Source: Nat Genet. 2025 Nov 18;57(12):3126–36. doi: 10.1038/s41588-025-02409-6 (PMC12695648; doi:10.1038/s41588-025-02409-6)
Supplement: Supplementary file 2 — Reporting Summary [file 41588_2025_2409_MOESM2_ESM.pdf]

nature portfolio

Corresponding author(s): Alex de Mendoza

Last updated by author(s): Apr 15, 2025

Reporting Summary

Nature Portfolio wishes to improve the reproducibility of the work that we publish. This form provides structure for consistency and transparency in reporting. For further information on Nature Portfolio policies, see our Editorial Policies and the Editorial Policy Checklist.

Statistics

For all statistical analyses, confirm that the following items are present in the figure legend, table legend, main text, or Methods section.

n/a	Confirmed	
☐	☒	The exact sample size (*n*) for each experimental group/condition, given as a discrete number and unit of measurement
☒	☐	A statement on whether measurements were taken from distinct samples or whether the same sample was measured repeatedly
☐	☒	The statistical test(s) used AND whether they are one- or two-sided<br>*Only common tests should be described solely by name; describe more complex techniques in the Methods section.*
☒	☐	A description of all covariates tested
☒	☐	A description of any assumptions or corrections, such as tests of normality and adjustment for multiple comparisons
☐	☒	A full description of the statistical parameters including central tendency (e.g. means) or other basic estimates (e.g. regression coefficient) AND variation (e.g. standard deviation) or associated estimates of uncertainty (e.g. confidence intervals)
☐	☒	For null hypothesis testing, the test statistic (e.g. *F*, *t*, *r*) with confidence intervals, effect sizes, degrees of freedom and *P* value noted<br>*Give P values as exact values whenever suitable.*
☒	☐	For Bayesian analysis, information on the choice of priors and Markov chain Monte Carlo settings
☒	☐	For hierarchical and complex designs, identification of the appropriate level for tests and full reporting of outcomes
☐	☒	Estimates of effect sizes (e.g. Cohen's *d*, Pearson's *r*), indicating how they were calculated

Our web collection on statistics for biologists contains articles on many of the points above.

Software and code

Policy information about availability of computer code

Data collection	Nanopore MinKNOW app was used to sequence the Nanopore libraries. This is the constantly updated version of Oxford Nanopore available to all users.
Data analysis	For sequence search and phylogenetic analysis HMMER3 (3.3.1), BLASTP(2.9.0+), MAFFT (7.475),trimAL (v1.4.rev15), IQ-TREE(2.1.2).<br>For Nanopore sequecing, basecalling and methylation analysis Guppy v6.5.7, modbam2bed (0.9.1), Dorado(0.7.2), Modkit (0.2.5), BEDTools (v2.29.2), UCSC bedGraphToBigWig, DeepTools2(3.5.0) and Bioconductor bsseq package for R were used.<br>For RNA-seq analysis HISAT2 (64-bit), Stringtie (2.1.2) and Kallisto (0.46.0) were used.<br>Fora ChIP-seq libary preparation and analysis fastp (0.20.0), bowtie2 (1.2), Sambamba (0.6.6), MACS2 (3.0.0a7) and DeepTools2 were used.<br>For genome assembly and re-annotation Guppy, Flye(v2.9-b1768), HyPo (v1.0.3), RagTag (v2.1.0), HISAT2 (2.2.1), Stringtie(v2.1.2), Trinity (v2.8.5), gmap (2020-10-14), Mikado (v2.3.0), Augustus (3.4.0), Liftoff (v1.6.3), PASA(v2.0.1), RepeatModeler2 (2.0.2a) and RepeatMasker (4.1.2-p1) were used.<br>The code used for the analysis of this paper can be found in https://github.com/AlexdeMendoza/6mA_evolution

For manuscripts utilizing custom algorithms or software that are central to the research but not yet described in published literature, software must be made available to editors and reviewers. We strongly encourage code deposition in a community repository (e.g. GitHub). See the Nature Portfolio guidelines for submitting code & software for further information.

Data

Policy information about availability of data

All manuscripts must include a data availability statement. This statement should provide the following information, where applicable:

- Accession codes, unique identifiers, or web links for publicly available datasets
- A description of any restrictions on data availability
- For clinical datasets or third party data, please ensure that the statement adheres to our policy

Nanopore raw sequencing data has been uploaded to ArrayExpress (https://www.ebi.ac.uk/arrayexpress/) under the accession number S-BSST1363, and the ChIP-seq data can be found in the GEO submission GSE261870. The ChIP-seq for C. reinhardtii was obtained from GSE59629, and T. vaginalis from GSE89662. Publicly available RNA-seq was obtained from PRJNA794325 (A. castellanii), PRJNA360056 (A. whisleri), SAMD00394225 (A. limacinum), PRJNA1091032 (C. perkinsii), PRJNA285347 (C. reinhardtii), PRJNA849385 (T. vaginalis), PRJNA262632 (C. limacisporum), PRJNA210187 (C. variabilis), GSE46692 (M. pusilla), GSE155535 (O. tauri), PRJNA642022 (N. fowleri), GSE249241 (A. parasiticum) and GSE68616 (C. fragrantissima). Annotation and other analysis files associated with this article can be found in: https://github.com/AlexdeMendoza/6mA_evolution. IGV genome browser session can be accessed for Creolimax fragrantissima (https://tinyurl.com/22zs884x), Spizellomyces punctatus (https://tinyurl.com/2d7jgzpz), Trichomonas vaginalis (https://tinyurl.com/2arl7o56), Acanthamoeba castellanii (https://tinyurl.com/22wjj72j), Naegleria gruberi (https://tinyurl.com/mr44r47h), Chlamydomonas reinhardtii (https://tinyurl.com/25aww7qk).

Research involving human participants, their data, or biological material

Policy information about studies with human participants or human data. See also policy information about sex, gender (identity/presentation), and sexual orientation and race, ethnicity and racism.

Reporting on sex and gender	n/a
Reporting on race, ethnicity, or other socially relevant groupings	n/a
Population characteristics	n/a
Recruitment	n/a
Ethics oversight	n/a

Note that full information on the approval of the study protocol must also be provided in the manuscript.

Field-specific reporting

Please select the one below that is the best fit for your research. If you are not sure, read the appropriate sections before making your selection.

☒ Life sciences ☐ Behavioural & social sciences ☐ Ecological, evolutionary & environmental sciences

For a reference copy of the document with all sections, see nature.com/documents/nr-reporting-summary-flat.pdf

Life sciences study design

All studies must disclose on these points even when the disclosure is negative.

Sample size	We sequenced all libraries to the highest coverage possible, aiming for >20x in all species, yet for some the gDNA was obtained from culture collections and due to pore clogging more limited amounts were obtained. This is explicit in the methods, figure legends and supplementary material.
Data exclusions	No data was excluded.
Replication	Technical replicates were obtained splitting in half the amount of reads for modification base-calling, and the results were compared to verify that each set of reads provided the same pattern.
Randomization	No randomization was needed.
Blinding	Blinding is not relevant to the study.

Reporting for specific materials, systems and methods

We require information from authors about some types of materials, experimental systems and methods used in many studies. Here, indicate whether each material, system or method listed is relevant to your study. If you are not sure if a list item applies to your research, read the appropriate section before selecting a response.

Materials & experimental systems

n/a	Involved in the study
☐	☒ Antibodies
☒	☐ Eukaryotic cell lines
☒	☐ Palaeontology and archaeology
☐	☒ Animals and other organisms
☒	☐ Clinical data
☒	☐ Dual use research of concern
☒	☐ Plants

Methods

n/a	Involved in the study
☐	☒ ChIP-seq
☒	☐ Flow cytometry
☒	☐ MRI-based neuroimaging

Antibodies

Antibodies used	Anti-Histone H3 (acetyl K27) antibody - ChIP Grade (abcam, Cat.No. ab4729) and Anti-trimethyl-Histone H3 (Lys4) Antibody (merckmilipore, Cat.No. 07-473) (Missing clone name and lot number), for western blot: Anti-Histone H3 (Abcam, ab1791), Histone H3K4me3 (Active Motif AB_2615077)
Validation	These antibodies have been commonly used in the Sebe-Pedros laboratory for profiling histone modifications in many species, and were selected for specificity and signal to noise ratio. Western blot antibodies are regularly used in Paul Hurd laboratory for working on honeybees.

Animals and other research organisms

Policy information about studies involving animals; ARRIVE guidelines recommended for reporting animal research, and Sex and Gender in Research

Laboratory animals	The study did not involve laboratory animals
Wild animals	The study did not involve wild animals
Reporting on sex	The study did not require any sex-based analysis as asexual single cell eukaryotes were used
Field-collected samples	The study did not involve samples collected from the field
Ethics oversight	No ethical approval was required as single cell eukaryotes were used

Note that full information on the approval of the study protocol must also be provided in the manuscript.

Plants

Seed stocks	n/a
Novel plant genotypes	n/a
Authentication	n/a

ChIP-seq

Data deposition

☒ Confirm that both raw and final processed data have been deposited in a public database such as GEO.

☐ Confirm that you have deposited or provided access to graph files (e.g. BED files) for the called peaks.

Data access links *May remain private before publication.*	https://www.ncbi.nlm.nih.gov/geo/query/acc.cgi?acc=GSE261870 Token: kdkbqsewtngrtaf
Files in database submission	raw fastq files and coverage bigwigs.

Genome browser session
(e.g. UCSC)

We provide 6 genome browser links: Creolimax fragrantissima: https://tinyurl.com/22zs884x
Spizellomyces punctatus: https://tinyurl.com/2d7jgzpz
Trichomonas vaginalis: https://tinyurl.com/2arl7o56
Acanthamoeba castellanii: https://tinyurl.com/22wjj72j
Naegleria gruberi: https://tinyurl.com/mr44r47h
Chlamydomonas reinhardtii: https://tinyurl.com/25aww7qk
.

Methodology

Replicates

Each ChIP-seq condition was obtained in duplicates (per histone mark & per species).

Sequencing depth

This is available in Supplementary Tables.

Antibodies

Anti-Histone H3 (acetyl K27) antibody - ChIP Grade (abcam, Cat.No. ab4729) and Anti-trimethyl-Histone H3 (Lys4) Antibody (merckmilipore, Cat.No. 07-473) (Missing clone name and lot number)

Peak calling parameters

macs3 callpeaks -t sample.bam -q 0.01 -g species_assembly_size

Data quality

Fraction of Reads in Peaks is >60% for all samples.

Software

All ChIP-seq data was analysed using fastp to trim the reads, and these were mapped to the genomes using bowtie2, allowing a maximum insert size of 2000 base pairs (-I 2000). Duplicate reads were removed using Sambamba. DeepTools2 was used to generate bigwig files and to visualise epigenomic data, as well as Integrative Genome Viewer.
