# Supplementary figures and images for: Adenine DNA methylation associated with transcriptionally permissive chromatin is widespread across eukaryotes
Source: Nat Genet. 2025 Nov 18;57(12):3126–36. doi: 10.1038/s41588-025-02409-6 (PMC12695648; doi:10.1038/s41588-025-02409-6)

Histone H3 antibody

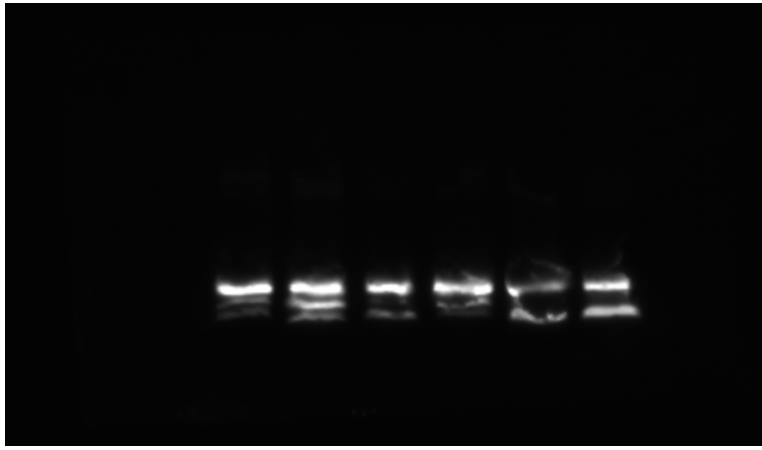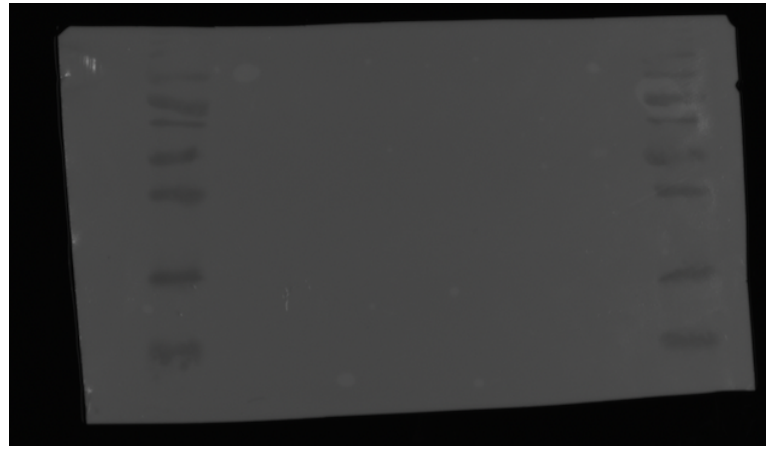

Histone H3K4me3 antibody

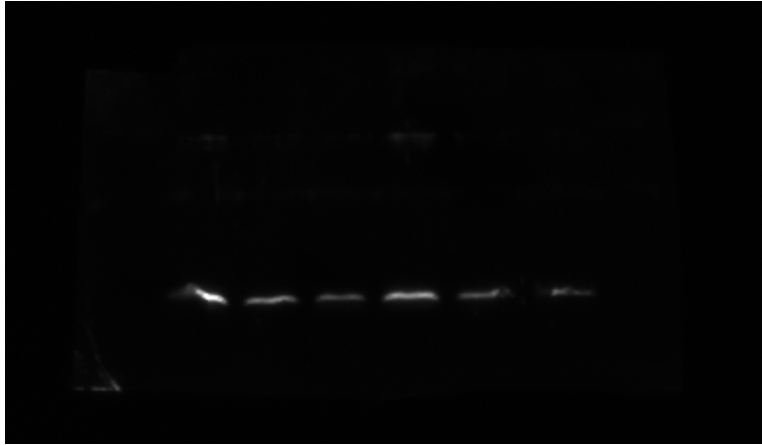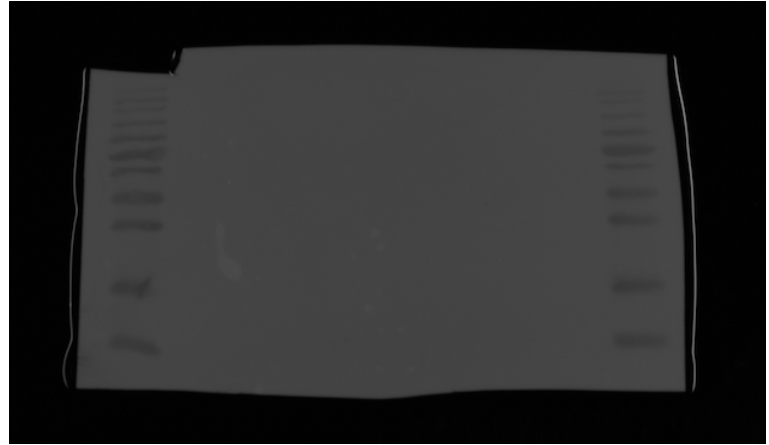

Supplement: Supplementary file 15 — Unprocessed western blots for Extended Data Fig. 10b. [file 41588_2025_2409_MOESM15_ESM.pdf]
